# Taxonomic Reassessment and Rediscovery of *Tulipa scardica* Bornm. in Greece: Insights from Integrated Analyses Compared to *T. undulatifolia* Boiss.

**DOI:** 10.3390/plants15091374

**Published:** 2026-04-30

**Authors:** Ioulietta Samartza, Eleni Kriemadi, Dimitris Pappas, Anastasia-Garyfallia Karagianni, Ioannis Kofinas, Theodora Matsi, Ioannis-Dimosthenis Adamakis, Georgios Tsoktouridis, Pepy Bareka, Nikos Krigas

**Affiliations:** 1 Department of Crop Science, Agricultural University of Athens, Iera Odos 75, GR-11855 Athens, Greece; isamartza@elgo.gr (I.S.); ekriemadi@aua.gr (E.K.); 2 Institute of Plant Breeding and Genetic Resources, Hellenic Agricultural Organization—Dimitra (ELGO-Dimitra), P.O. Box 60458, GR-57001 Thessaloniki, Greece; kofinas.kallergis@gmail.com (I.K.); gtsok@elgo.gr (G.T.); 3 Institute of Nuclear & Radiological Sciences and Technology, Energy & Safety (INRASTES), National Centre for Scientific Research “Demokritos”, GR-15310 Agia Paraskevi, Greece; d.pappas@ipta.demokritos.gr; 4 Soil Science Laboratory, School of Agriculture, Aristotle University of Thessaloniki, GR-54124 Thessaloniki, Greece; anastasia-asia@hotmail.com (A.-G.K.); thmatsi@agro.auth.gr (T.M.); 5 Department of Biology, National and Kapodistrian University of Athens, GR-15784 Athens, Greece; iadamaki@biol.uoa.gr; 6 Wild Tulip Specialist Group, IUCN Species Survival Commission, International Union for the Conservation of Nature, IUCN Headquarters, Rue Mauverney 28, 1196 Gland, Switzerland; 7 Institute of Olive Tree, Subtropical Crops and Viticulture, Hellenic Agricultural Organization—Dimitra (ELGO-Dimitra), GR-71307 Heraklion, Greece; 8 Department of Agriculture, School of Agricultural Sciences, Hellenic Mediterranean University, GR-71410 Heraklion, Greece

**Keywords:** integrative taxonomy, morphology, karyology, SEM, DNA barcoding, phylogenetic analysis, Liliaceae

## Abstract

*Tulipa scardica* (Balkan endemic) was last recorded in Greece in 1896, possibly attributed to longstanding taxonomic ambiguity, as it has frequently been considered as conspecific with *T. gesneriana* or *T. undulatifolia*. In the present study we aimed to investigate the historical Greek locality of *T. scardica* and to reassess its taxonomic status in comparison with the closely related *T. undulatifolia* (also native to Greece and member of *T. scardica* complex). Targeted field surveys were conducted to verify the presence of *T. scardica* in Greece. The newly identified tulip population was subjected to an integrated analytic approach, including qualitative and quantitative morphological assessment, seed micromorphology, DNA barcoding, karyological investigation, and habitat/soil properties analyses. These datasets were comparatively evaluated against four reference populations of *T. undulatifolia*. Molecular data did not provide consistent species-level resolution, whereas morphological and karyological evidence statistically supported their distinction. Mitotic metaphase chromosomes of *T. scardica* were documented herein for the first time, while first-time scanning electron microscopy (SEM) analysis revealed the presence of different types of stomatal complexes in seed coats of both taxa. In addition, soil parameters differed between the examined taxa, and those of the rediscovered population were consistent with habitat preferences of *T. scardica*. Although both taxa exhibited considerable variability, the combined evidence derived from the present study strongly supported the rediscovery of *T. scardica* in Greece after approximately 130 years, unless proven otherwise in a wider context across its Balkan range.

## 1. Introduction

*Tulipa scardica* Bornm. is a rare Balkan endemic species of the genus *Tulipa* L. (Liliaceae, Tribe Tulipae Duby; [1]) distributed primarily in southeastern Kosovo, North Macedonia, and northern Greece [2]. The species was originally described in 1923 from a small population in southern former Yugoslavia and was named after Mount Scardus (Šar Mountains), the region where it was first discovered [3]. Its distribution is restricted to the southwestern Balkans, including areas near the village Krivenik in southern Kosovo close to the North Macedonian border, as well as the Vardar and Pčinja valleys and limited parts of the high mountain pastures of the Šar Mountains in North Macedonia [4] (https://florafaunafun.com/endangered-species-in-north-macedonia/#google_vignette, accessed on 1 February 2026). Owing to its narrow distribution range and small population size (e.g., only a few dozens of individuals occurring in less than 1 km^2^ in Kosovo), *T. scardica* has been assessed as Critically Endangered and is legally protected in Kosovo [5], as an endangered species in the Republic of North Macedonia (https://florafaunafun.com/endangered-species-in-north-macedonia/#google_vignette, accessed on 1 February 2026), and as a Vulnerable one in the recent Greek Red List (https://redlist.necca.gov.gr/en/assessment/?id=224613851, accessed on 3 April 2026).

In Greece, *T. scardica* has been only historically recorded, and no confirmed subsequent observations exist despite its legal protection under the Greek Presidential Decree 67/1981. The only herbarium record documenting its presence in Greece dates back to 1896 [6]; this unique specimen (P. Sintenis 1555, 16 May 1896; https://herbarium.emg.umu.se/record.php?AccessionNo=1307482&Page=1&AaccNr=1307482&Ainst=LD&Acoll=&Aid=143602619&nrRecords=1&ARecord=1, accessed on 1 February 2026) is currently deposited in the Lund Herbarium (L!) and it was taxonomically revised and confirmed as *T. scardica* by K. Persson (det./conf. K. Persson, 2002; https://herbarium.emg.umu.se/record.php?AccessionNo=1307482&Page=1&AaccNr=1307482&Ainst=LD&Acoll=&Aid=143602619&nrRecords=1&ARecord=1, accessed on 1 February 2026). The locality has been respectively georeferenced (Lat 39.65 Long 21.616667; precision ca. 2000 m).

*Tulipa scardica* faces significant conservation challenges due to its limited distribution and small population sizes across its range [5]; it inhabits thermophilous woodlands in mountainous regions of the southwestern Balkan Peninsula, often associated with serpentine substrates, although earlier studies indicate that it is rather characteristic of limestone habitats [7]. The species exhibits considerable morphological variability among populations, including differences in leaf morphology, flower colour, filament length, and anther characteristics. Individuals typically bear pale red flowers with violet external markings, although variation in floral colouration has been observed [7]. *Tulipa scardica* constitutes the nominal species of the “*T. scardica* complex”, a group of closely related taxa including *T. serbica* Tatic & Krivošej, *T. albanica* Kit Tan & Shuka, *T. kosovarica* Kit Tan, Shuka & Krasniqi, and *T. luanica* Millaku [8,9]. These taxa are morphologically similar, and often difficult to distinguish in the field, leading to ongoing taxonomic debate regarding their status as distinct species or as intraspecific variants [9,10,11]. Since its original description, *T. scardica* has been treated as a distinct species in major botanical works such as “The genus *Tulipa*: Tulips of the world” [12] and the “Prodromus florae peninsulae Balcanicae” [13]. In contrast, Flora Europaea [14] considers it as a synonym of *T. boetica* Boiss. & Heldr. while the Aegean Flora [15] treats the *T. boetica* Boiss. & Heldr. as a synonym of *T. undulatifolia* Boiss. Furthermore, Flora Europaea [14] regards *T. undulatifolia* and *T. scardica* as conspecific whereas other studies have synonymized *T. scardica* under *T. gesneriana* L. [5,9]. Preliminary phylogenetic studies provide partial support for the recognition of *T. scardica* and *T. albanica* as distinct species, and recent studies acknowledge the existence of a taxonomically challenging *T. scardica* complex [8,9]. Nevertheless, molecular resolution within the broader complex remains limited, highlighting the need for further cytotaxonomic investigations to achieve a more robust taxonomic framework [9]. 

Within this context, integrative taxonomy has emerged as a powerful approach that extends traditional morphology-based species delimitation. By combining multiple lines of evidence—including molecular, ecological, and distributional data—it enables the formulation of more accurate species hypotheses, accounting for cryptic diversity, phenotypic plasticity, and hybridization, factors that are often overlooked when relying solely on morphological criteria [16,17,18,19].

Within this framework, karyotype analyses are widely recognized as a valuable tool in cytotaxonomy, contributing to species delimitation and the reconstruction of phylogenetic relationships. Recent studies on karyotype evolution increasingly integrate classical cytology with molecular, morphological, and biogeographic data, enhancing our understanding of genome organization, chromosomal evolution, and their relationships with phenotypic expression and evolutionary processes [20,21]. Karyotypic heterogeneity together with morphological, systematic, and molecular characteristics is considered a fundamental criterion for the classification of plant taxa. The karyotype describes the number, size, and morphology of the chromosome complement of an individual, population or a species as observed under the microscope and reflects the cumulative effects of structural and organizational changes acting on the respective genomes [22]. The members of the genus *Tulipa* specifically are characterized by a basic chromosome number of x = 12, a feature consistently supported to date by numerous cytological studies [23,24,25,26,27,28]. Across the genus members, an extensive variability of ploidy levels has been documented to date, from diploid (2x) to hexaploid (6x), with some *Tulipa* species exhibiting more than one ploidy level. This variability has raised questions regarding the extent to which ploidy level alone provides sufficient and reliable insight into the evolutionary history of the members of this genus [29,30]. Tulips generally possess karyotypes that are relatively symmetrical in chromosome size, dominated by submetacentric and acrocentric chromosomes. Distinctive structural features such as satellites and secondary constrictions are only inconsistently observed. Although several efforts have been made to classify *Tulipa* species based on chromosome morphology [31,32,33], these approaches have shown limited success due to the overall similarity of their karyotypes. Within the *T. scardica* complex, the chromosome number for *T. scardica* was determined as 2*n* = 2x = 24 by previous studies [34,35]. Subsequently, other studies [36] have estimated the nuclear DNA amount of 2C = 68 pg as diploid from a specimen of *T. scardica* from North Macedonia. For *T. undulatifolia*, a diploid chromosome number of 2*n* = 2x = 24 was previously reported [37] originating from five Greek wild-growing populations (Phocis, Attiki, Korinthia, Argolis, and Arkadia regions) and a microphotograph of a karyotype has also been provided from a population occurring in the Attiki (Attica) region. Other studies have reported the same chromosome number 2*n* = 2x = 24 for this species [38,39], with the latter additionally presenting a karyotype microphotograph based on wild-growing material from Turkey. Although the chromosome number 2*n* = 3x = 36 has been reported for *T. undulatifolia* both in the abstract and in the chromosome data table of the previous study [38], this count appears to be erroneous. The caption of the karyotype microphotograph as well as the karyogram given in the same study clearly indicates a chromosome number of 2*n* = 2x = 24, which is consistent with the 24 chromosomes undoubtedly visible in the published image. This internal discrepancy likely resulted in the subsequent erroneous inclusion of the chromosome number 2*n* = 3x = 36 for *T. undulatifolia* in the Chromosome Counts Database (CCDB, version 1.66). However, although another study confirmed diploidy (2*n* = 2x = 24) [36], this study further reported a triploid population from Greece (Dídima, Peloponnese) with 2*n* = 3x = 36 chromosomes.

DNA barcoding serves as another powerful molecular tool in plant taxonomy, using standardized nuclear and plastid DNA regions like *rbc*L, *mat*K, *trn*H-*psb*A, and ITS2 to enable species identification [40]. The phylogeny of tulips has been studied widely over the years [8,9,41,42,43], aiming to disentangle the relationships between closely related taxa. *Tulipa scardica* has been found to form a *T. scardica* complex clustering tightly in subgenus *Tulipa* with combined markers providing the strongest resolution over single loci [9]. Members of this so-called “scardica complex”, including *T. scardica*, *T. serbica*, *T. albanica*, *T. kosovarica*, and *T. luanica*, have been genetically studied [8]; the latter study synonymized *T. luanica* and *T. kosovarica* with *T. serbica* and supported the distinction between *T. scardica* and *T. albanica* [9]. Even though the latter study offered valuable insight into the *T. scardica* complex, the need for more specialized tools is highlighted to fully resolve species boundaries. On the other hand, *T. undulatifolia* has been studied genetically in several works, e.g., [41,42,44], and shows genetic similarity to yet differentiation from *T. scardica* [42].

Morphological traits including seed morphology remain fundamental tools for species delimitation. It is known that fruits and seeds represent a critical stage in the plant life cycle, functioning not only as protective structures or carriers for the embryo but also as dynamic organs that regulate dormancy, water uptake, and gas exchange, all of which may influence the species’ reproductive success [45]. Central to these processes is the seed coat, which acts as both a physical barrier and a regulatory interface mediating environmental signals that influence seed metabolism, dormancy release, and germination timing [46]. Although stomata are traditionally regarded as leaf-specific structures, their sporadic occurrence on seed coats has been documented to date in several angiosperm lineages, where they may contribute to water permeability and gas exchange during seed development and germination [47,48,49,50]. In this context, seed coat micromorphology—including epidermal cell patterning, surface sculpturing, and specialized pores—has gained recognition as a valuable source of taxonomic and evolutionary insight, particularly in bulbous and geophytic taxa in which species-specific traits often reflect ecological specialization [51,52,53,54,55]. Despite this noteworthy potential, seed structural traits in monocotyledons remain comparatively underexplored to a large extent [56]. Nevertheless, relatively recent studies on *Tulipa* seed morphology [55,57] and responses to stratification regimes [58,59] and germination success [60,61] emphasize the importance of integrating anatomical, physiological, and developmental approaches to better understand the germination ecology and to support conservation strategies for rare and endemic taxa. Collectively, such findings underscore the complex interplay between seed coat structure and function and render scanning electron microscopy as another powerful tool employed in the elucidation of possible interspecific differences with diagnostic significance regarding closely related taxa.

From an ecological perspective, wild-growing plant population structure is shaped by a complex interaction of environmental factors, among which climate and edaphic conditions play a particularly critical role. Soil properties in particular not only influence species distribution [62] but also their physiological performance and adaptive potential [63,64]. The natural variation in nutrient availability and the balance of essential macro- and micro-elements provide valuable insights into the growth dynamics, developmental strategies, and ecological adaptation of different species [63,64,65]. Across the diverse phytogeographical regions of Greece, tulip species exhibit distinct morphological and ecological traits that reflect long-term adaptation to local habitat conditions [63]. Consequently, soil properties can be expected to reveal population- and species-specific responses.

Beyond biogeographic and taxonomic interest, *T scardica* represents an important component of regional plant genetic resources, particularly as a range-restricted local Balkan endemic species which is a wild relative of cultivated tulips (crop wild relative). The wild-growing members of the genus *Tulipa* encompass considerable genetic diversity that may support new tulip breeding programmes targeting traits such as disease resistance, flower morphology, and environmental adaptation in the face of climate crisis. However, the effective conservation and sustainable use of this diversity depend on accurate species delimitation. Taxonomic uncertainty—such as the treatment of *T. scardica* as conspecific with *T. undulatifolia*—may lead to the under-representation or loss of biologically distinct genetic resources, undermining in situ conservation prioritization and hindering ex situ management efforts. Clarifying the taxonomic status of *T. scardica* is therefore essential for preserving its genetic integrity and diversity, and facilitating its inclusion both in conservation management schemes and new breeding initiatives.

In this complex taxonomic context and importance framework, the aims of the present study were to: (i) investigate and confirm the occurrence of *T. scardica* in Greece based on historical records and contemporary field surveys; (ii) evaluate whether *T. scardica* differs morphologically, anatomically, ecologically, and/or genetically from the frequently regarded conspecific *T. undulatifolia* belonging to the *T. scardica* complex; and (iii) provide a comprehensive characterization of *T. scardica* using an integrative framework that includes morphological, anatomic, karyotypic, edaphic, and molecular data. This integrative taxonomic approach was adopted to yield more robust and reliable insights, particularly for taxonomically challenging species groups such as the *T. scardica* complex.

## 2. Results

### 2.1. Quantitative and Qualitative Morphology

A total of 140 individuals were examined in situ for quantitative traits (Table 1), including 25 individuals of *T. scardica* and 115 individuals of *T. undulatifolia* originating from four populations (38 from Fana, 16 from Emporiós, 44 from Dídima, and 17 from the Agios Stefanos population). For each species, means and standard deviations were calculated, followed by Kruskal–Wallis tests and Dunn’s post hoc comparisons. Most measured variables revealed statistically significant differences between species means (Table 1).

Overall, *T. scardica* comprised generally smaller plants, typically bearing three leaves. Morphologically, *T. scardica* exhibited longer leaves and wider tepals than *T. undulatifolia*, whereas the latter was characterized by a filament-to-anther ratio of less than 1 (Table 1).

**Figure 1:**
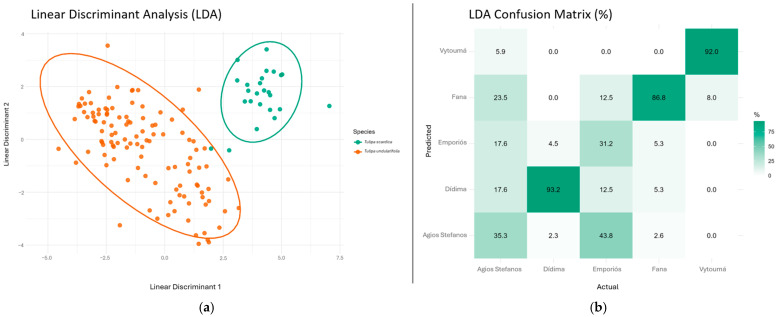
(**a**) Linear Discriminant Analysis (LDA) of quantitative morphological traits showing separation between *Tulipa scardica* (green points) and *T. undulatifolia* (orange points). Each point represents an individual plant plotted along the first two linear discriminant axes (LD1 and LD2). Ellipses indicate the 95% confidence regions around the group centroids for each species. The separation of the two clusters along LD1 reflects distinct multivariate morphological differentiation between the taxa. (**b**) Confusion matrix (%) from Linear Discriminant Analysis (LDA) based on jackknifed (leave-one-out) classification of individuals among populations. Values represent the percentage of individuals from each actual population (columns) assigned to each predicted population (rows). Colour intensity increases with classification percentage.

A Linear Discriminant Analysis (LDA) was performed to explore morphological variation among the five populations studied. The following traits were included in the analysis: plant length, stem width, length/width of lowest leaf, length/blotch of outer tepal, length/width of outer tepal, length of outer tepal/length of inner tepal, width of outer tepal/width of inner tepal, length/blotch of inner tepal, length/width of inner tepal, length of inner filament/length of anther and length of outer filament/length of anther. Linear Discriminant Analysis (LDA) based on standardized morphological traits revealed a separation between *T. scardica* and *T. undulatifolia* along the first discriminant axis (LD1), with minimal overlap between species confidence ellipses. The second discriminant axis primarily captured within-species variability, which was notably greater in *T. undulatifolia* than in *T. scardica*. When LDA was performed using populations as grouping factors, populations of *T. undulatifolia* clustered together and were clearly separated from *T. scardica* (Figure 1a).

The jackknifed LDA classification showed variable discrimination among populations (Figure 1b). Classification success was highest for *T. undulatifolia* from Dídima (93.2%), *T. scardica* from Vytoumá (92.0%), and *T. undulatifolia* from Fana (86.8%), indicating strong morphological differentiation. In contrast, *T. undulatifolia* from Emporiós (31.2%) and Agios Stefanos (35.3%) showed lower classification success, reflecting overlap with other populations. Misclassification patterns revealed particularly strong overlap between Agios Stefanos and Emporiós, as well as partial overlap of Fana with Vytoumá and other populations.

**Figure 2:**
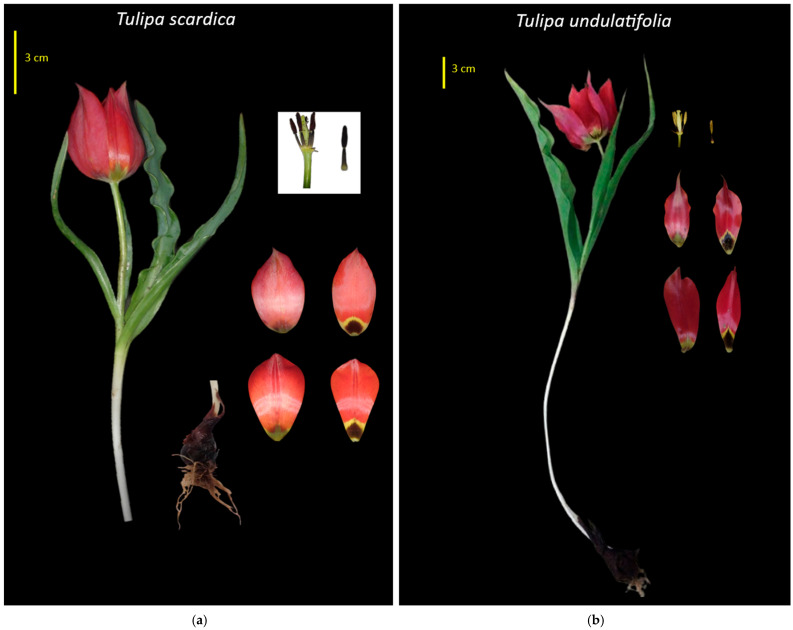
Photographs of the fresh plant structures of (**a**) *Tulipa scardica* and (**b**) *T. undulatifolia* (Dídima population). Respective scale bars appear in yellow.

Regarding the non-numeric traits (Figure 2), the two species exhibited a high degree of overall morphological similarity. All populations had individuals with pubescent stems, without stolons, linear-lanceolate lowest and second lowest leaves that exhibited undulation at various extents. The flowers were campanulate, and bicoloured, namely bright red/scarlet, with a dark blotch covered with a yellow strip on the outer part. Across the five *Tulipa* populations examined—*Tulipa scardica*, *T. undulatifolia* Dídima, *T. undulatifolia* Fana, *T. undulatifolia* Emporiós–Komi, and *T. undulatifolia* Agios Stefanos—overall morphology was highly conserved, although several traits allowed meaningful differentiation (Figure 2).

Both species shared the presence of stem pubescence and absence of stolons. Bulbs bore a brown, papery tunic, slightly hairy at the base. Leaf morphology was remarkably uniform: the lowest and second-lowest leaves were consistently linear-lanceolate with undulation at various extents in both positions. In all populations, flowers were campanulate and bore a bicoloured basal blotch, consisting of a dark to black central area bordered by a distinct yellow margin. Populations of *T. undulatifolia* exhibited flowers with a pronounced “waist” as described in previous studies [12]. Inner and outer tepal colouration—bright red internally and pale red externally—was also consistent across the populations (Figure 2). Both taxa shared glabrous inner tepal apexes and filaments coloured brownish-black to purplish-black. Filament bases lacked pubescence across both species, providing no distinguishing value.

More diagnostic differences were observed in tepal shape and tepal-apex morphology. *Tulipa scardica* exhibited an elliptic outer tepal with an apex ranging from acute to obtuse, distinguishing it from all *T. undulatifolia* populations, which displayed narrowly elliptic outer tepals with acute to acuminate apexes. The inner tepals further separated *T. scardica*: they were obovate to spathulate with an obtuse apex. In contrast, the *T. undulatifolia* populations were characterized by elliptic-ovate inner tepals narrowed at the base, consistently ending in an acute apex (Figure 2).

Anthers of *T. scardica* appeared yellowish or—in most cases—purplish, whereas in *T. undulatifolia* they were mostly yellow. Pollen in *T. scardica* seemed purplish whereas in *T. undulatifolia* it appeared greenish-yellow (Figure 2).

### 2.2. Seed Morphology and SEM

A total of 30 seeds from each species was evaluated for both qualitative and quantitative seed traits. The overall seed morphology, i.e., shape, colour, and embryo morphology, showed high similarity among populations. However, significant differences were detected between species for several quantitative traits (Table 2; Supplementary Materials Figure S1). Overall, *Tulipa scardica* exhibited smaller seeds (total and seed size), smaller seed wings, but more developed embryos compared to *T. undulatifolia* (Table 2). Seeds from both species exhibited sector seed shape of brown colour with a linear, visible embryo. Seeds (*n* = 30) of *T. scardica* from Vytoumá and *T. undulatifolia* from Dídima weighed 0.1488 g and 0.1580 g, respectively.

**Figure 3:**
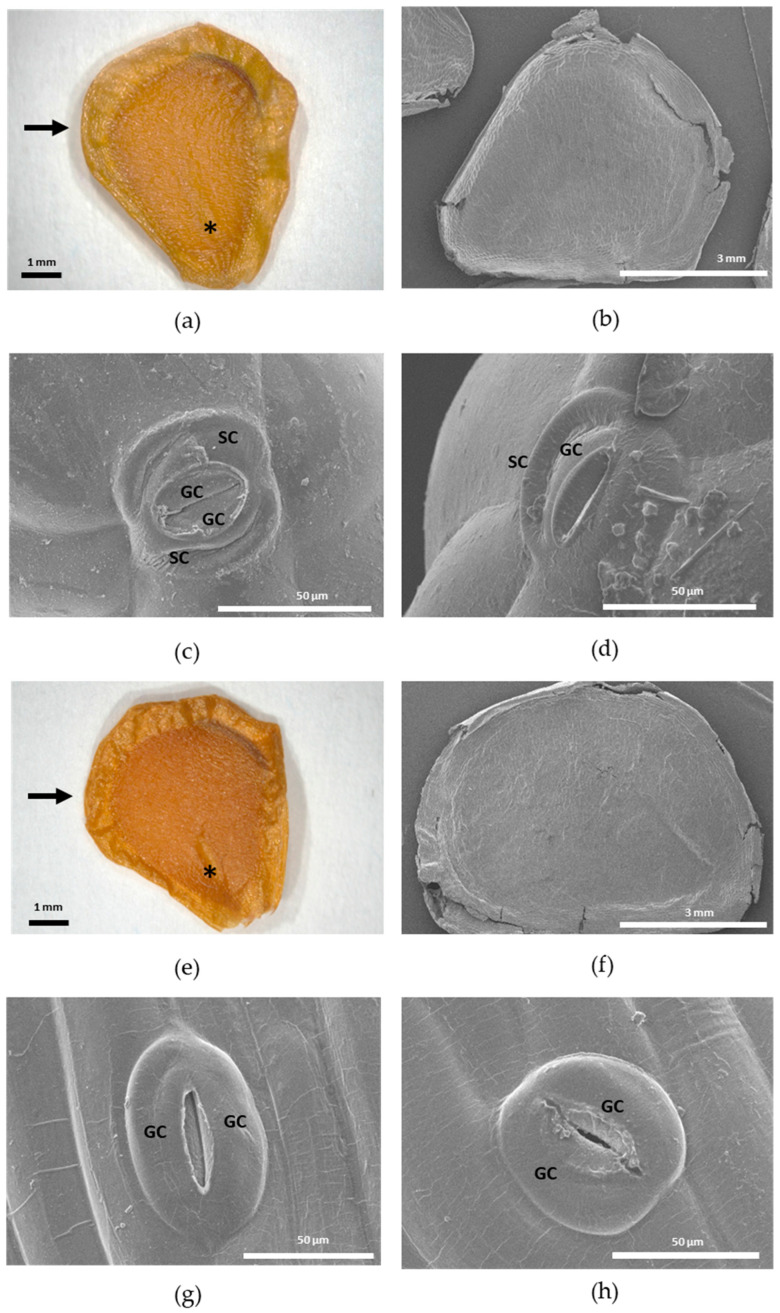
Seed morphology and micro-structures of *Tulipa scardica* (**a**–**d**) and *T. undulatifolia* (**e**–**h**). *Tulipa scardica*: (**a**) Whole seed in lateral view under stereomicroscope; (**b**) Seed surface observed by scanning electron microscopy (SEM); (**c**,**d**) SEM details of stomatal complexes with guard cells (GCs) bordered with subsidiary cells (SCs). *Tulipa undulatifolia:* (**e**) Whole seed in lateral view under stereomicroscope; (**f**) Seed surface observed by SEM; (**g**,**h**) SEM details of the stomatal complexes and guard cells (GCs). Asterisks indicate embryo position, and arrows testa margin. Scale bars: as depicted (1 mm in (**a**,**e**); 3 mm in (**b**,**f**); 50 µm in (**c**,**d**,**g**,**h**)).

Seeds of *T. scardica* and *T. undulatifolia* showed similar overall morphology but differed in stomatal organization on the seed coat. In both species, seeds were flattened and asymmetrical, with a broadly ovate outline and a smooth to slightly uneven surface under stereomicroscopy (Figure 3a,e). SEM observations revealed a compact testa with subtle surface relief and marginal folding in both taxa (Figure 3b,f).

Distinct stomata were observed on the seed coat of both species. In *T. scardica*, the stomatal complexes were well developed and consisted of two elongated guard cells enclosing a distinct stomatal aperture (Figure 3c,d). These guard cells were surrounded by clearly differentiated subsidiary cells, forming a complete stomatal complex. The subsidiary cells were morphologically distinct from adjacent epidermal cells and appeared to provide structural support to the guard cells.

In contrast, stomata in *T. undulatifolia* consisted mainly of paired guard cells delimiting a narrow aperture (Figure 3g,h). Subsidiary cells were indistinct or not clearly differentiated from the surrounding epidermal cells. The guard cells in this species appeared more symmetrical and less deeply sunken into the seed coat surface compared to those of *T. scardica*. These observations indicate clear interspecific differences in the structure and complexity of stomatal complexes on the seed surface.

### 2.3. Karyology

**Figure 4:**
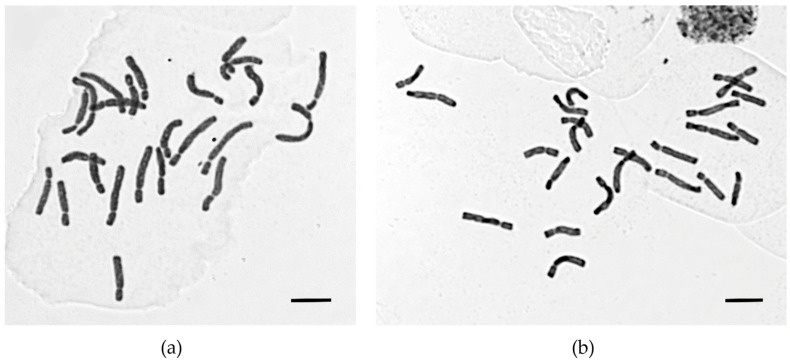
Microphotographs of mitotic metaphase plates of the studied *Tulipa* species: (**a**) *T. scardica*, 2*n* = 2x = 24; (**b**) *T. undulatifolia*, 2*n* = 2x = 24. Scale bars = 10 μm.

The chromosome number was determined as 2*n* = 2x = 24 for both *T. scardica* and *T. undulatifolia*, across all studied populations. Karyotypes were symmetrical, consisting of submedian and subterminal (sm and st) chromosome pairs. The karyotype formula of *T. scardica* is 2*n* = 2x = 8 sm + 4 sm-SAT + 2 sm/st-SAT + 10 st, whereas that of *T. undulatifolia* is given as 2*n* = 2x = 12 sm + 4 sm-SAT + 6 sm/st + 2 st-SAT. Microphotographs of metaphase plates are shown in Figure 4, while the karyotype morphology and karyotype indices are provided in Table 3 and Table 4.

Chromosome sizes ranged from 8.54 μm, representing the smallest chromosome pair, to 13.91 μm, representing the largest pair in *T. scardica*, and from 8.53 μm to 15.05 μm in *T. undulatifolia*. The average chromosome length (ACL) was 11.09 μm and 11.47 μm, respectively, for the two species. The total chromosomes’ length was 266.07 μm for *T. scardica*, and 275.35 μm for *T. undulatifolia*.

Regarding the coefficient of variation in chromosome length, CV_CL_, the lowest value of the variation was observed in *T*. *scardica* (15.92), whereas 19.22 was recorded in *T. undulatifolia*. The coefficient of variation in the centromeric index (CV_CI_) was 24.95 for *T. scardica* and 23.07 for *T. undulatifolia*. The mean centromeric asymmetry (MCA) was 45.73 and 45.67 for *T. scardica* and *T. undulatifolia*, respectively.

Welch’s *t*-test confirmed statistically significant differences between the two species for CV_CL_ (t = −4.807, *p* = 0.00014) and CV_CI_ (t = 2.713, *p* = 0.012). Effect size analysis (Cohen’s d) revealed very large differences for CV_CL_ (d ≈ 1.574) and CV_CI_ (d ≈ 0.833). In contrast, no statistically significant differences were detected for Min l+s (*p* = 0.972), Max l+s (*p* = 0.081), TCL (*p* = 0.404), ACL (*p* = 0.352), THL (*p* = 0.357), or M_CA_ (*p* = 0.948). Although a moderate effect size was observed for Max l+s (d ≈ 0.72), this difference was not statistically significant, while all other parameters exhibited small or negligible effect sizes (d < 0.35). In this context, the large Cohen’s d values underscore the substantial effect sizes, highlighting the biological relevance of these differences even with the smaller sample size of *T. scardica*.

Satellite chromosomes were detected in both studied species. These satellites were typically located at the distal ends of the long chromosome arms and were usually small and spherical. However, in both taxa the satellites were small and not always clearly visible, likely due limitations of the squash technique used for chromosome preparation. In *T. undulatifolia*, one to three pairs of SAT chromosomes were observed, which were either submetacentric (sm-SAT) or submetacentric/acrocentric (sm/st-SAT) or a combination of both. In *T. scardica*, the maximum number of satellite chromosomes observed in the studied population comprised two pairs of submetacentric (sm-SAT) chromosomes and one submetacentric/acrocentric (sm/st-SAT) chromosome pair.

### 2.4. DNA Barcoding

Multiple barcode regions were sequenced including ITS, *trn*L/*trn*F, *psb*A/*trn*H, and one additional cpDNA locus namely NADH-plastoquinone oxidoreductase subunit 3 (*ndh*C gene). A total of 24 sequences were produced in this study and were submitted to GenBank (NCBI), assigned unique accession numbers (Table S2).

The alignment of ITS (alignment length = 655 bp) indicated only two divergent loci: position 543, mainly influencing *T. albanica*, and position 555, showing inconsistency across taxa (A/G; Table S3). The *trn*H-*psb*A marker (alignment length = 455 bp) also exhibited high levels of conserved regions. Specifically, samples U2 and U3 showed a gap at locus 24, while U1 and U2 displayed SNPs at loci 60 and 380. *Tulipa albanica* samples [8] showed SNPs at loci 93, 95, 96, and 98 (Table S4).

The alignment of the *trn*L/*trn*F marker (alignment length = 780 bp) revealed more divergent regions. At loci 113–122 and 707–711, multiple A substitutions were observed, whereas at loci 290 and 344, *T. albanica*, *T. scardica*, and *T. undulatifolia* shared the same SNPs. Moreover, one sample of *T. scardica* (T15, [8]) showed an SNP at locus 319; at locus 508, the taxa examined in this study exhibited an additional A; S4 showed a gap at locus 623; and some samples of *T. kosovarica* presented an SNP at locus 679 (Table S5).

In contrast, the alignment of *ndh*C (alignment length = 897 bp) indicated a less conserved and more differentiated cpDNA region. In total, 44 variable loci/regions were detected among taxa. This higher variability may be attributed to the fact that the taxa analyzed for this particular marker were not as closely related as those examined above, which all belong to the *T. scardica* complex (Table S6).

**Figure 5:**
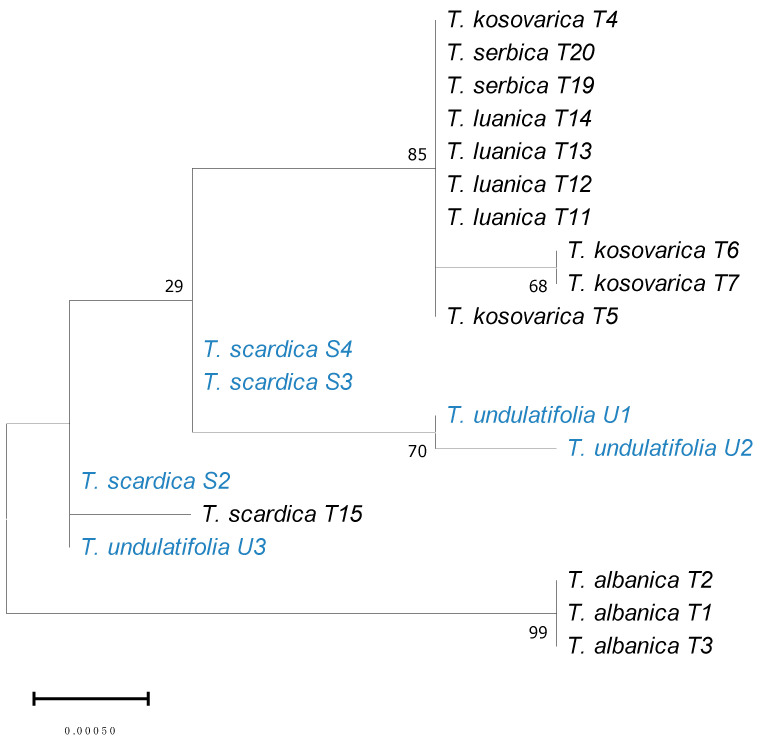
Phylogenetic tree resulting from the ITS + *trn*L/*trn*F + *psb*A/*trn*H molecular markers among *Tulipa* taxa included in the *T. scardica* complex inferred from DNA sequence data. Bootstrap support values are indicated at the nodes. Branch lengths are proportional to the number of substitutions per site. The samples studied herein are indicated in blue. All accession numbers used and produced for phylogenetic analysis are reported in Table S2. Evolutionary history was inferred using the Maximum-Likelihood method. The optimal tree is shown. The evolutionary distances were computed using the Tamura 3-parameter method.

The phylogenetic tree (Figure 5) constructed for ITS + *trn*L/*trn*F + *psb*A/*trn*H markers included species sequences from the *T. scardica* complex studied in previous studies [8] (GenBank accession numbers are provided in Table S2). This phylogenetic analysis resulted in several well-defined clades corresponding largely to currently recognized species. *Tulipa albanica* formed a strongly supported and genetically distinct clade (bootstrap = 99), with minimal divergence between its sampled individuals [8]. Samples of *T. kosovarica*, *T. luanica*, and *T. serbica* clustered in a broader group, although relationships among these taxa received moderate bootstrap support (Figure 5), indicating limited resolution of their internal branching order. *Tulipa scardica* samples, including S4 which is identified as *T. scardica*, grouped together, with the individuals (S3 and S4) forming a weakly supported subclade, while S2 and T15 [8] showed slight divergence within the same group. *Tulipa undulatifolia* also formed a consistent group (U1–U2), closely related to the *T. scardica* clade. Branch lengths were generally short across the tree (scale = 0.00050), indicating low overall genetic divergence among taxa.

**Figure 6:**
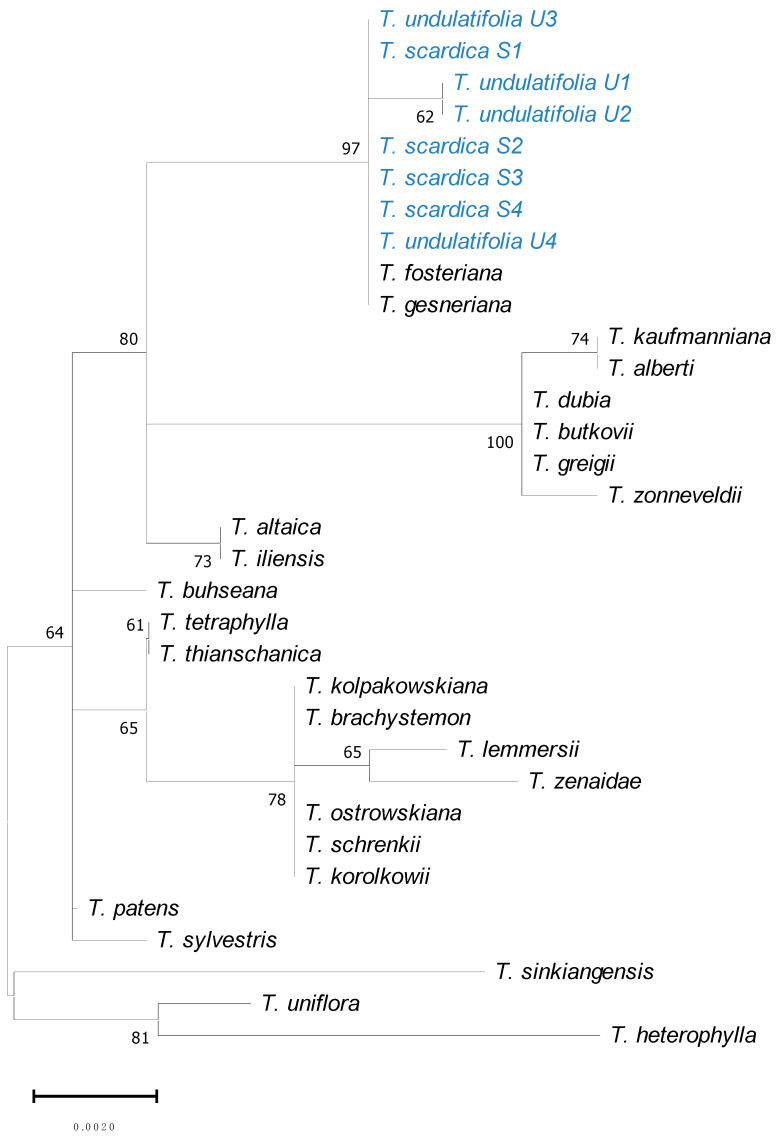
Phylogenetic tree resulting from the *ndh*C molecular marker among *Tulipa* taxa inferred from DNA sequence data. Bootstrap support values are indicated at the nodes. Branch lengths are proportional to the number of substitutions per site. The samples studied herein are indicated in blue. All accession numbers used and produced for phylogenetic analysis are reported in Table S2. inferred by using the Maximum Likelihood method. The evolutionary distances were computed using the Tamura 3-parameter method.

The phylogenetic analysis for *ndh*C (Figure 6) showed *T. scardica* as a well-supported clade nested within a larger group that also included *T. undulatifolia*, *T. fosteriana* W. Irving, and *T. gesneriana*. All individuals of *T. scardica* (S1–S3 and S4) clustered together without deep internal variation, indicating low intraspecific genetic differentiation for this marker. The *T. scardica* clade was positioned closely to *T. undulatifolia*, which formed a subclade (bootstrap = 62), and this combined lineage received high support (bootstrap = 97, Figure 6). This broader group was separated from additional Eurasian taxa, forming successive, moderately to strongly supported clades. Overall branch lengths were short (scale = 0.0020), suggesting relatively low genetic divergence across the genus for this particular molecular marker.

### 2.5. Soil Properties

The physicochemical characteristics and available nutrient concentrations of soils from the four populations are presented in Table 5. The soil of *T. scardica* was sandy loam, alkaline in reaction, non-calcareous, with relatively high cation exchange capacity (Table 5) [66]. Organic C was higher in this soil compared with the other sites, resulting in a C/N ratio of 12.1 (Table 5), which falls within the typical range reported for arable soils (8–15, with a mean value of 12) [66]. Similarly, all soils from *T. undulatifolia* were alkaline in reaction and had medium texture (sandy loam or loam), but Dídima 2 and Emporiós soils, with loamy texture, had considerably higher clay contents and CEC values, and markedly elevated Ca concentrations (Table 5).

With respect to fertility status, the soil of *T. scardica* appeared low in terms of the primary macronutrients (N–P–K) (Table 5). Specifically, available NO_3_–N was below commonly accepted sufficiency levels (10 mg kg^−1^) [67] and exchangeable K was marginally sufficient [68], whereas available P was above the lowest sufficiency value of 10 mg kg^−1^ [69]. Among secondary macronutrients, Mg was particularly high (Table 5). Regarding micronutrients, Fe and Mn were present at high levels relative to standard sufficiency ranges, while Cu, Zn and B (Table 5) were within the sufficiency ranges. According to previous studies, the sufficiency ranges in mg kg^−1^ are 2.5–5.0 for Fe, 1.0–5.0 for Mn, 0.1–2.5 for Cu, 0.2–2.0 for Zn, and 0.1–2.0 for B [70]. Comparable patterns were observed among the other sites, although all *T. undulatifolia* soils exhibited notably higher K, Ca, and B concentrations, while P, Mg and Fe were higher in *T. scardica* soil. Overall, substantial differences in soil texture, chemical properties, and nutrient availability were recorded among the four soils.

## 3. Discussion

### 3.1. Significance of Morphological Evidence

Species delimitation is often challenging due to the complex nature of biodiversity and evolutionary processes [17]. Morphology-based taxonomy often faces obstacles, such as phenotypic plasticity, convergent evolution, and cryptic species [71]. Consequently, higher taxonomic resolution is often achieved through integrative taxonomy, that combines traditional morphological data with molecular, karyological, and ecological data, providing a more accurate and comprehensive framework for species identification and classification [17,19].

In the present study, a total of 140 individuals (25 individuals of *T. scardica* and 115 individuals from four populations of *T. undulatifolia*) were first-time examined in situ fresh for quantitative morphological traits, leading to statistically significant differences among species (Table 1). Overall, *T. scardica* exhibited markedly smaller plants, almost half the size of *T. undulatifolia*, characterized by narrower stems bearing primarily three leaves (Table 1). Although leaf shape was similar between species, *T. scardica* exhibited longer and narrower leaves, whereas *T. undulatifolia* had relatively broader ones. Floral traits were also of diagnostic value: *T. scardica* had shorter but wider tepals, a proportionally larger basal blotch relative to tepal length, and filaments that were longer than the anthers, in contrast to *T. undulatifolia* where filament length was consistently shorter than anther length. Moreover, anthers were significantly smaller in *T. scardica* individuals.

LDA clearly separated *T. scardica* and *T. undulatifolia*, with minimal overlap between species confidence ellipses. As anticipated, within-species morphological variability was notably greater in *T. undulatifolia* (however, anticipated) than in *T. scardica*. When populations were used as grouping factors, LDA maximized among-population differences; however, substantial overlap among *T. undulatifolia* populations was observed, indicating that morphological similarity remains high even among geographically distant populations. These results suggest that although population-level structure is present, it is weak compared to shared morphology and species-level similarity does not prevail. The high grouping success (Figure 1b) of the Vytoumá population supported its morphological distinctiveness, which was consistent with its initial assignment to *T. scardica*. In contrast, the extensive overlap among the remaining populations suggested weak morphological structuring within *T. undulatifolia*.

In contrast to the quantitative morphological characters examined, the categorical traits showed somehow lower discriminatory power between the two taxa and appeared to be more conserved between the two taxa. This outcome was expected, as earlier treatments and descriptions of these species have often been ambiguous, even treating *T. scardica* as a synonym of *T. undulatifolia* [14]. Moreover, *T. scardica* exhibited considerable morphological variability, further complicating its delimitation [7,10,11].

Originally, *T. scardica* was described as a uniflorous bulbous plant with ovate bulbs enclosed in brown tunics and a glabrous stem about 20 cm tall. It bears three (rarely four) very long, narrow leaves, slightly to distinctly undulate. The flowers are medium-sized and bell-shaped, with nearly equal tepals bearing a conspicuous black basal blotch broadly margined with yellow. The stamens have black filaments and yellow anthers [3,13]. Other descriptions of *T. scardica* [12] generally follow the original one, adding that plants are typically shorter (around 10 cm tall), with 3–4 basal leaves at ground level, and that the stamens have black filaments with black to brown anthers and blackish-purple pollen [12]. Other studies, e.g., [7,10,11], report greater variation in leaf number (3–7), leaves that are flat to weakly undulate and generally larger, and variable perianth colours, excluding pure yellow with a yellow base and occasionally a dark blotch [10,11]. According to such descriptions, filaments may be black or white, and anthers yellowish to violet-purple [10,11]. The population of *T. scardica* examined herein conforms well to the original description made by Bornmüller in 1923 and subsequent accounts [12,13], while also encompassing part of the broader variability reported in subsequent studies. In addition, our results indicate that *T. scardica* and *T. undulatifolia* differ mainly in tepal shape and tepal-apex morphology, and in overall flower form, *T. undulatifolia* consistently exhibit a characteristic “waisted” flower shape [12]. Additional distinguishing features include anther and pollen colour: *Tulipa scardica* has purplish pollen, whereas *T. undulatifolia* has greenish-yellow pollen.

The inclusion of additional populations undoubtedly attributable to *T. scardica* from its Balkan range would further strengthen the robustness of the conclusions of the current study, as the investigation of merely one population from its Greek range exhibits a significant limitation. However, the species is extremely rare in Greece, not reported from elsewhere within the national territory. Therefore, multiple populations of *T. undulatifolia* (four populations) were included for comparative purposes, as the latter species is well documented and more widely distributed in Greece. Future studies incorporating additional populations across the species’ broader south-western Balkan range will be essential to further refine its taxonomic delimitation within the *T. scardica* complex.

Compared to numerical data, it is concluded that the main traits that delimit these two species are specific floral traits, i.e., the shape of tepals and the apex of the tepals and the colour of the anthers. Leaf shape is also a delimiting trait. Moreover, it should be noted that the studied population of *T. scardica* exhibits remarkably smaller individuals in height than the four studied populations of *T. undulatifolia.*

### 3.2. Findings Based on Seed Morphology and SEM

Although several studies have suggested that seed morphology alone may be insufficient as a primary diagnostic character for species discrimination [72,73,74], this approach can undoubtedly provide informative and complementary evidence for species differentiation. In some cases, particularly among closely related taxa, seed morphology has proven to be a reliable and consistent source of taxonomic information. For example, in *T. uniflora* (L.) Besser ex Baker and *T. heteropetala* Ledeb., seed morphology was identified as the most stable and informative trait for species identification [57]. Building on such findings, an identification key was developed for tulip species in the East Kazakhstan region based on seed characters; however, the authors noted that no single seed trait was sufficient to distinguish all species at the same time [57].

In the present study, the seed morphology of *T. scardica* and *T. undulatifolia* was described for the first time. Qualitative SEM observations revealed noticeable variability among populations; however, these patterns should be interpreted cautiously, as they may reflect population-level variation or environmental influences. Confirmation of the observed trends would require examination of a broader sampling range, replication across multiple populations, and assessment over different temporal scales which are beneficial to minimizing potential environmental effects.

A particularly noteworthy finding was the first-time documentation of stomata on the seed coat of both *T. scardica* and *T. undulatifolia*. Although seed coat stomata are relatively uncommon, their sporadic occurrence across angiosperms has been documented previously [48,75]. In *Swietenia macrophylla* King (Meliaceae), for instance, seed stomata were shown to be structurally complete, consisting of two prominent guard cells frequently accompanied by subsidiary cells, remaining permanently open at maturity, with the highest densities observed in the embryo region [48].

While stomatal densities were not quantified in the present study, SEM imaging revealed comparable qualitative structural features in the examined *Tulipa* species. In *T. scardica*, stomata were clearly differentiated, comprising paired guard cells surrounded by well-defined subsidiary cells, closely resembling the stomatal complexes described for *S. macrophylla*. In contrast, *T. undulatifolia* exhibited simpler stomatal complexes, in which subsidiary cells were weakly differentiated or indistinguishable from adjacent epidermal cells. Similar interspecific variation in stomatal complexity has previously been attributed to differences in epidermal developmental pathways rather than to differences in physiological function [48,75].

The functional significance of seed coat stomata remains a subject of debate. Experimental work on *S. macrophylla* demonstrated a strong correlation between stomatal presence and water uptake during imbibition, with dye-tracer experiments indicating preferential water entry through stomatal apertures rather than through the surrounding epidermis [48]. Although such functional assays were not conducted for *Tulipa* members, the observation of permanently open stomata with distinct guard-cell morphology suggested that these structures may similarly represent regions of reduced resistance within the testa.

In addition to facilitating water uptake, seed coat stomata have been proposed to contribute to gas exchange during seed development, particularly in non-photosynthetic seeds surrounded by thick integuments [75,76]. Despite the absence of active stomatal movement, the large pore size and association with intercellular air spaces may allow sufficient diffusion of respiratory gases to the developing embryo [48]. In the examined *Tulipa* species, the persistence of stomata on the mature seed coat likely reflects developmental continuity between the ovular epidermis and the testa, with stomatal differentiation maintained even after most testa cells become non-living.

Support for the physiological relevance of seed coat stomata has also been provided by other studies in members of the genus *Iris* (Iridaceae), where four out of seven examined species exhibited stomata on the seed coat [49]. In these species, stomata varied in shape, size, and frequency, were generally much less abundant than leaf stomata, and most remained permanently open. The authors suggested that such stomata may play roles in seed development, testa formation, dormancy, and water uptake during germination. This interpretation is consistent with the observations made in the present study in the studied members of the genus *Tulipa* and supports the view that—even infrequent or structurally simplified—seed coat stomata may contribute to physiological processes essential for successful germination.

From a systematic perspective, the interspecific differences observed between *T. scardica* and *T. undulatifolia*, particularly regarding the presence or absence of clearly differentiated subsidiary cells, represent stable micromorphological features. As reported for *S. macrophylla* where stomatal distribution correlates with testa thickness [48], such qualitative architectural traits may provide additional diagnostic characters for distinguishing closely related taxa. Consequently, seed coat stomatal architecture may hold value not only for understanding aspects of seed development but also as a complementary character set for species delimitation within members of the genus *Tulipa*. This research line should be considered in future taxonomic studies related to the genus *Tulipa*.

### 3.3. Findings Based on Karyological Data

This study confirms previous reports for the chromosome number of 2*n* = 2x = 24 in *T. scardica* and *T. undulatifolia*. Although microphotographs of mitotic metaphase plates have previously been published for *T. undulatifolia*, this is the first time that they are provided for *T. scardica*. In addition, a comprehensive karyotypic analysis for both species is presented, including the estimation of all indices related to intra- and interchromosomal asymmetry, as well as the formulation of karyotype formulas for each taxon.

Several differences in karyotype morphology were detected between the two species. *Tulipa undulatifolia* exhibited a greater proportion of submetacentric chromosomes. The higher CV_CL_ value recorded for *T. undulatifolia* reflected increased interchromosomal asymmetry, indicating more pronounced differences in chromosome size compared to *T. scardica*. In contrast, nearly identical M_CA_ values in both species suggested a conserved pattern of intrachromosomal asymmetry that was associated with centromere position. The slightly lower CV_CI_ in *T. undulatifolia* further indicated reduced variability in centromere positions among its chromosomes. Differences in chromosome size range, average chromosome length (ACL), total chromosomal length (TCL), and total haploid chromosomal length (THL) were not pronounced between the two species. Collectively, these results suggested that karyotypic differentiation between the two species was driven primarily by variation in chromosome length rather than by changes in centromere position.

Overall, karyotype analyses revealed herein that *T. scardica* and *T. undulatifolia* are distinguishable based on their cytogenetic patterns, though their divergence remains subtle. *Tulipa undulatifolia* was characterized by a higher proportion of submetacentric chromosomes and a greater interchromosomal asymmetry (CV_CL_), whereas both species showed similar levels of intrachromosomal asymmetry (M_CA_), with slightly lower centromere variability (CV_CI_) in *T. undulatifolia*. Differences between the largest and smallest chromosome pairs were relatively small. This limited divergence is consistent with the well-established stability of the basic chromosome number in *Tulipa* (x = 12) and with the cytological evolutionary trends observed in members of this genus. The chromosome size in tulips tends to change in a relatively uniform manner along both arms of the same chromosome, through the addition of similar amounts of DNA to each chromosome regardless of chromosome length, leading to reduction in both inter- and intrachromosomal asymmetry (i.e., CV_CL_, CV_CI_) [28]. Consequently, although statistically supported differences were detected in this study, pronounced karyotypic differentiation between the two species would not be expected, and the observed differences likely represent a subtle yet taxonomically informative level of cytogenetic divergence. The magnitude of these differences further supported their biological relevance despite the smaller sample size. These findings aligned with the observed karyotype morphology, suggesting that overall chromosome size characteristics remained largely conserved, while indices reflecting chromosomal variability and centromere positioning showed subtle divergence.

### 3.4. Observations from DNA Barcoding

The genetic placement of the wild-growing *T. scardica* according to a phylogenetic tree constructed from a concatenated alignment of ITS + *trn*L/*trn*F + *psb*A/*trn*H (Figure 5) molecular markers highlighted both genetic distinctiveness and internal structuring. The separation of S2 from the S3/S4 subclade might suggest individual-level differentiation. Its close relationship with *T. undulatifolia* indicates a shared evolutionary history. In contrast to the weaker resolution observed among *T. kosovarica*, *T. luanica*, and *T. serbica*, *T. scardica* seems to be more structured within the tree, differing clearly from other species of the complex, yet showing similarity with *T. undulatifolia*. Similarly, the *ndh*C marker indicated the placement of *T. scardica* within a larger clade containing *T. fosteriana* and *T. gesneriana* (Figure 6), confirming broader evolutionary connections among these taxa as described previously [4,8]. However, several nodes in the phylogeny showed weaker support, thus limiting confident interpretation of relationships. It is worth noting that, in the current study, the analysis was mainly focused on the ITS + *trn*L/*trn*F + *psb*A/*trn*H markers, aiming to produce comparable results to previous studies [9]. Thus, the *ndh*C marker worked more as a supplementary marker; however, sequence data for this region were scarce and largely restricted to chloroplast DNA sequences available in GenBank. As a result, it was not possible to include this marker in a concatenated analysis without substantially reducing taxon sampling.

In general, it is argued that the genetic closeness of *T. scardica* to *T. undulatifolia* does not necessarily indicate conspecificity, as the two studied taxa are closely related and belong to the same subgenus (namely Subgenus *Tulipa* [77]) and complex (*T. scardica* complex), thus sharing a high degree of similarity. Comparable limitations have been reported in previous studies using universal molecular markers [9,42], highlighting the need for the development of new, more informative molecular tools or markers for resolving relationships among closely related taxa.

Notably, the material from North Macedonia referred to as *T. scardica* (Sample S4) represents an identification performed in the frame of ex situ maintenance of plant displays in a botanic garden (Gothenburg botanic garden). However, we were not able to independently verify its taxonomic identification, as no live material was available for detailed examination. For this reason, S4 was treated with caution and considered as putatively belonging to the *T. scardica* complex rather than as a definitively identified specimen.

As species delimitation based solely on molecular evidence would be disconnected from the species’ history, the integration of morphological and ecological data is essential for a comprehensive taxonomic assessment. Further molecular studies are therefore required to investigate population structure and genetic differentiation between the two taxa studied herein.

### 3.5. Insights from Soil Properties and Habitat Preferences

The present study provides the first characterization of the soil properties associated with *T. scardica*, comparing them with three soil samples from *T. undulatifolia* populations (Dídima 1, Dídima 2, and Emporiós). Although variability among soils was evident (Table 5), several traits were common to both taxa. In general, soils of all populations were alkaline. The soil of *T. scardica* was classified as sandy loam, with a C/N ratio comparable to that of typical cultivated soils. Similarly, one population of *T. undulatifolia* (Dídima 1) also occurred on sandy loam soil, whereas the other two populations were established on loamy soils with higher clay content and CEC.

In terms of fertility, the soil of *T. scardica* was characterized by inadequate available NO_3_–N, sufficient available P, and marginally sufficient K. The three *T. undulatifolia* soils likewise showed inadequate NO_3_–N and adequate micronutrient concentrations, similar to those of *T. scardica*. However, clear differences were observed between soils. All *T. undulatifolia* soils exhibited lower C/N ratios and lower available P concentrations than *T. scardica*, while available K levels were higher—reaching excessive levels in the loamy soils of Dídima 2 and Emporiós. These findings indicate that, despite broadly similar alkaline reaction and medium texture, the two taxa were associated with soils differing in nutrient balance, particularly with respect to P and K availability.

The habitat of most populations of *T. undulatifolia* studied herein (Dídima, Agios Stefanos, Emporiós) is abandoned cultivations and/or olive groves, while the Fana population was growing on a rocky slope. *Tulipa scardica* was also found on a rocky slope.

Most populations of *T. undulatifolia* examined in this study (Dídima, Agios Stefanos, Emporiós) were found in semi-natural habitats resulting from agricultural abandonment, mainly former cultivations and old olive groves. These areas are typically characterized by open, low-growing herbaceous vegetation, showing reduced management and grazing pressure. In contrast, the population at Fana was located on a rocky slope with sparse plant cover, exposed bedrock, and shrub vegetation. A similar habitat was observed for *T. scardica*, which was also recorded on a slope with scattered trees and shrubs. In contrast to many tulip species that frequently occur in former agricultural land, *T. scardica* was not recorded in such habitats. The population was found in openings (elevation range: 326–332 m a.s.l.) within shrubby vegetation dominated by *Quercus coccifera* L., *Juniperus oxycedrus* L. and phrygana on a northeast-facing slope developed over serpentine rocky substrates. Limestone outcrops were present at the base of the hills and along the ridges of Koziakas Mountain (also known as Mt. Kerketio), forming the western margin of the Thessalian plain. Associated herbaceous perennial species recorded in the habitat include the Balkan endemics *Colchicum haynaldii* Heuff., *Fritillaria thessala* Boiss., and *Odontarrhena heldreichii* (Hausskn.) Španiel, Al-Shehbaz, D.A.German & Marhold. These findings corroborate earlier reports suggesting that *T. undulatifolia* is primarily associated with abandoned agricultural land and rocky slopes [11,12,13], whereas *T. scardica* shows a stronger specialization for dry, rocky habitats [3,7,13,14,15].

### 3.6. Combination of Evidence

Based on the combination of evidence, the present study suggests overall that *T. scardica* and *T. undulatifolia* are two distinct species differing mainly in morphology, seed traits, and karyotype, despite lacking clear genetic separation based on DNA barcoding or consistent occurrence in clearly different habitats with different soil properties. Evidently, *Tulipa scardica* is smaller, with longer and narrower leaves, wider but shorter tepals, with a filament-to-anther ratio greater than one, purplish pollen, and smaller seeds with narrower wings and a more developed embryo, which are equipped with well-defined stomatal complexes on the seed coat. In contrast, *T. undulatifolia* is larger, has wider leaves, longer tepals, with a filament-to-anther ratio below one, yellow to greenish pollen, larger seeds with wider wings and a less developed embryo, and weaker seed coat stomatal organization; it also shows a more asymmetric karyotype (Table 6).

From a phytogenetic resources perspective, the distinction between *T. scardica* and *T. undulatifolia* carries important conservation implications. Morphologically, karyologically, genetically, and/or ecologically distinct taxa such as *T. scardica*, that was studied herein, may contain unique adaptive traits and gene sets, thus representing irreplaceable components of biodiversity. If *T. scardica* was not re-established in this study as a distinct biological entity and remained an ambiguous entity or was taxonomically submerged under *T. undulatifolia*, its potentially distinct gene pool due to restricted distribution range and ecological specificity may have been overlooked in conservation planning. By providing integrative evidence supporting the species status for *T. scardica*, the present study highlights the need for targeted conservation actions, including germplasm representativity in ex situ conservation facilities and in situ habitat protection. The latter emphasizes that accurate taxonomy can serve as a critical factor for the conservation and sustainable use of plant genetic resources across scales, particularly for taxa with unconsolidated status, limited distribution, legal protection, and high conservation concern such as *T. scardica*. To this end, the present study offered additional back-up material of accessioned seeds and bulbs currently maintained under ex situ conservation in Greece (GR-1-IPBGR-25,175; GR-1-IPBGR-25,176; GR-1-IPBGR-25,409). This back-up material maintained under ex situ conservation represents valuable living specimens of random wild genotypes from *T. scardica*’s southernmost distribution limit, which increase by 33,3% the total species representativity in botanic gardens and seedbanks worldwide according to the PlantSearch tool of the Botanic Gardens Conservation International (see online BGCI’s PlantSearch at https://plantsearch.bgci.org/taxon/67126, accessed on 3 April 2026).

## 4. Materials and Methods

### 4.1. Study Areas and Sampling

**Figure 7:**
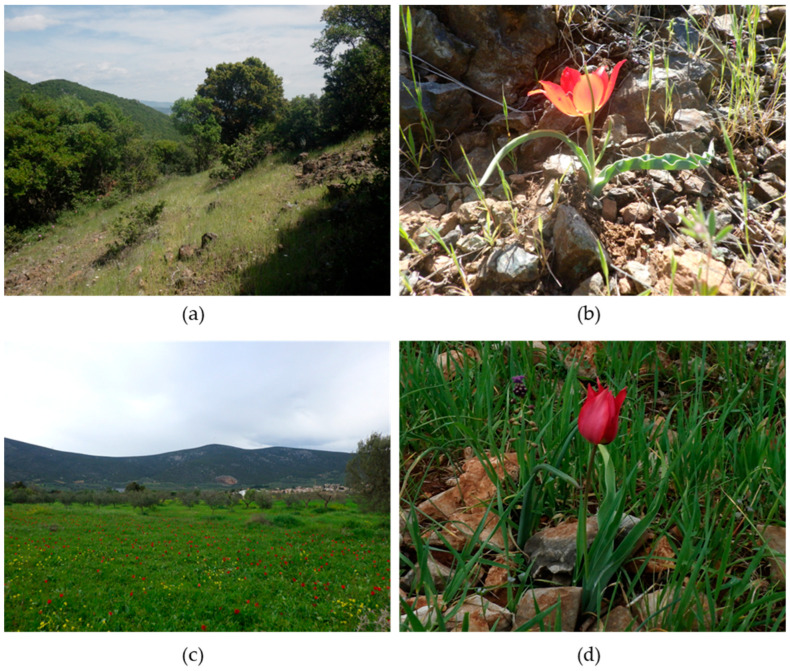
Habitat and plant individuals of the studied populations of *Tulipa scardica* (**a**,**b**) and *T. undulatifolia* ((**c**,**d**); Dídima population).

Field trips were organized during summer 2024 and spring and summer 2025 to visit the wild-growing populations of the tulip species investigated and study the morphological traits in situ. The exploration area for *Tulipa scardica* was determined after discussion with local inhabitants from the wider region of Trikala and Kalabaka, Greece, the area where it was originally recorded in 1896 (Figure 1a, Box S1, [6]). Due to the morphological resemblance and possible confusion of *T. scardica* with *T. undulatifolia* [14], four populations of the latter were visited across Greece, namely in Agios Stefanos, Attika (N38.163056, E23.870278), Dídima, Argolida, Peloponnese (Figure 7, N37.450361, E23.177306), Fana, Chios Island (N38.2108, E25.93555) and Emporiós -Komi, Chios Island (N38.203178, E26.029964), to achieve proportionality of the species’ morphological variation and to exclude possible occurrence of *T. scardica* in these areas. The in situ measurements and the collection of living wild-growing plant material were performed using special collection permits issued by the competent Greek authorities (e.g., YPEN/DPD/80381/5557 of 9 August 2024 and YPEN/DPD 73029/4891 of 1 July 2025). The herbarium specimens and/or the living plant materials (dormant bulbs) of *T. scardica* and *T. undulatifolia* are deposited in the Herbarium of the Agricultural University of Athens (Herbarium acronym ACA) and/or are currently maintained ex situ in the Balkan Botanic Garden of Kroussia (Herbarium acronym BBGK), Institute of Plant Breeding and Genetic Resources of the Hellenic Agricultural Organization Demeter in Thessaloniki, Greece. Different samples from the collected tulip species in the wild were assigned to a unique IPEN (International Plant Exchange Network) accession number (Supplementary Materials, Box S2).

Although the population examined in the present study belongs to the *T. scardica* complex and, aside from *T. scardica*, could potentially be assigned to either *T. albanica* due to geographical proximity with Albania where it is a local endemic [11] or *T. suaveolens* Roth (syn. *T. schrenkii* Regel), which is often used as a comparative species in studies of the *T. scardica* group [11], these alternatives were excluded. First, *T. albanica* differs markedly from *T. scardica* in key morphological traits, particularly in the colouration of the perianth segments [11]. Second, *T. schrenkii* exhibits a different geographical distribution that does not comprise the study area [11]. Therefore, considering the strong morphological resemblance of the examined population to *T. scardica* and due to previous reports of this species in the region [6], we considered its assignment to *T. scardica* to be the most conceivable.

### 4.2. Morphological Traits

In each of the above-mentioned wild-growing populations visited per taxon, the individuals were evaluated both for quantitative and qualitative traits (Supplementary Materials Table S1) using diagnostic characteristics of previously published studies in members of the genus *Tulipa* [39]. For the specific plant parts calculations, a digital ruler and a calliper (Mitutoyo Europe GmbH, Neuss, Germany) were employed to record all the respective measurements.

### 4.3. Seed Morphometry and Scanning Electron Microscopy

A total of 30 mature seeds from *T. scardica* and *T. undulatifolia* (Supplementary Materials Figure S1) were evaluated for seven seed morphological parameters following previously published studies [55,72]. Specifically, the measured traits (abbreviation; units) were: total length (TL; mm), total width (TW; mm), embryo length (EL; mm), seed length (SL; mm), seed width (SW; mm), wing width (WW; mm), and the distance from the crossing point of TL and TW to the nearest edge of the seed (CPL; mm). In addition, the ratios EL/SL, TL/TW, SW/WW, SL/SW, and TL/CPL were calculated. The weight of 30 seeds from each population was also measured. Seeds were further evaluated for qualitative traits, including shape, colour, and embryo morphology [55]. Observations were conducted using a stereomicroscope (Euromex, Duiven, The Netherlands), and photographs were taken with the macro lens of a RICOH WG-6 (Ricoh Imaging Company Ltd., Tokyo, Japan) compact camera.

For Scanning Electron Microscopy (SEM), samples were fixed in 4% (*w*/*v*) paraformaldehyde in PEM buffer (50 mM PIPES, 5 mM EGTA, 5 mM MgSO_4_, pH 6.8) for 1 h. Fixed samples were then washed with PEM and dehydrated using a graded ethanol series until absolute ethanol. Ethanol was eventually removed and hexamethyldisilazane (HMDS) was added to the dehydrated samples, which were left to dry overnight. Prior to imaging, dry samples were mounted on an appropriate SEM stub with adhesive carbon tape and coated with a 5 nm thick layer of Au/Pd (80/20), using a Leica EM ACE200 (Leica Microsystems GmbH, Wetzlar, Germany) sputter coater at 20 mA. Imaging was performed under a Hitachi FlexSEM 1000 II (Hitachi High-Tech Corporation, Tokyo, Japan) in secondary electron (SE) mode, at 7 kV accelerating voltage. Observations were also performed using a Stemi 2000-C stereomicroscope (ZEISS, Jena, Germany) equipped with an integrated ProgRes C3 digital camera (JENOPTIK AG, Jena, Germany).

### 4.4. Statistical Analysis

Quantitative morphological traits were analyzed using multivariate statistical approaches. Multivariate analysis of variance (MANOVA) was applied to test for overall differences in mean morphology among groups. Linear Discriminant Analysis (LDA) was performed on standardized morphological variables to examine patterns of morphological differentiation among specimens. LDA was conducted using population as the grouping factor to evaluate the extent to which morphological variation is structured among populations. Because LDA maximizes among-group variance relative to within-group variance, overlap among populations in discriminant space was interpreted as evidence of morphological similarity despite geographic separation. In addition, LDA results were visualized with specimens coloured by species to assess whether species-level differentiation persisted independently of population structure. A confusion matrix was generated based on the LDA results using leave-one-out (jackknifed) cross-validation, summarizing the percentage of individuals correctly and incorrectly classified into predefined groups.

All statistical analyses were conducted in R. Data handling, cleaning, and selection of informative variables were performed using *dplyr* [78] and *tidyr* [79]. Before the multivariate analyses, the quantitative variables were centred and scaled to unit variance using base R functions to ensure comparability among traits. Linear Discriminant Analysis (LDA) was carried out using the MASS package [80] to evaluate differentiation, and the resulting discriminant scores were visualized with *ggplot2* [81], with species-level confidence ellipses produced using *ggplot2* and *factoextra* [82]. To assess overall multivariate differences among species, a MANOVA based on Wilks’ lambda was implemented. For univariate comparisons, the *rstatix* [83] package was used to perform Kruskal–Wallis tests followed by Dunn’s post hoc pairwise comparisons with Benjamini–Hochberg adjustment, while *multcompView* [84] was employed to generate compact letter displays (CLDs) summarizing significant differences among populations.

All seed measurements were obtained using ImageJ v.1.54r software [85]. Statistical analyses were performed in R v.4.4.2 to test data normality, apply transformations when necessary, and conduct *t*-tests.

### 4.5. Karyology

Chromosome numbers were determined from metaphase plates obtained from root tip meristems following a squash preparation protocol based on previous studies [86]. Seven populations of *T. undulatifolia* and one from *T. scardica* were studied (Supplementary Materials Box S3). Root tips were initially pretreated for six hours in a 1:1 solution of 8-hydroxyquinoline (0.003% *w*/*v*) and colchicine (0.1% *w*/*v*), followed by fixation in Carnoy’s solution (absolute ethanol: glacial acetic acid, 3:1 *v*/*v*) for 24 h at 0–4 °C. Subsequently, the material was hydrolysed in 1 N HCl at 60 °C for 12 min and stained with Feulgen reagent [87] for approximately 1 h. Between five and ten karyotypes per population were analyzed using a Zeiss Axiophot photomicroscope (ZEISS, Jena, Germany), with a Jenoptik Gryphax camera (JENOPTIK AG, Jena, Germany).

Chromosome morphology and nomenclature were assigned according to [88,89,90], incorporating the recommendations of [91,92,93].

Karyomorphometric analyses were performed by calculating the following parameters: maximum and minimum chromosome lengths, total chromosome length (TCL), average chromosome length (ACL), and total haploid length (THL) of the chromosome complement. In addition, interchromosomal asymmetry (CV_CL_; [94], intrachromosomal asymmetry (M_CA_; [95,96,97]), and the coefficient of variation in the centromeric index (CV_CI_; [94,97]) were calculated to assess centromere position heterogeneity. For both tulip species, the chromosome complement was characterized by measuring the lengths of the long and short arms of homologous pairs, total chromosome length, r-index, centromeric index, arm difference ratio, and relative length (R-length).

Statistical analyses were conducted to compare karyomorphological parameters (Min l+s, Max l+s, TCL, ACL, THL, CV_CL_, MCA, CV_CI_) between *T. scardica* (*n* = 10) and *T. undulatifolia* (*n* = 36). Given the unequal sample sizes and heterogeneity of variances, comparisons were conducted using Welch’s *t*-test, for its robustness to these conditions. Statistical significance was determined at the 0.05 level (*p* < 0.05).

In addition to *p*-values, effect sizes were calculated using Cohen’s d to quantify the magnitude of differences between the two species, interpreted according to conventional thresholds: small (d = 0.2), medium (d = 0.5), large (d = 0.8), and very large (>1.2).

### 4.6. DNA Barcoding

Samples from four populations of *T. undulatifolia* were examined: U1, *T. undulatifolia* Dídima [42]; U2, *T. undulatifolia* Agios Stefanos; U3, *T. undulatifolia* Fana; and U4, *T. undulatifolia* Emporiós. In addition, three samples from the studied population of *T. scardica* (S1, S2, and S3) were analyzed, together with S4, a taxonomically verified sample of *T. scardica* originating from North Macedonia [42].

Young leaves were collected in spring 2025 for DNA analysis and were stored prior to analysis at −20 °C. DNA extraction, PCR amplification, reagents, sequencing of PCR products, and subsequent DNA sequence analyses were performed following the methodology and tools outlined by our own previous studies [42,98]. Oligonucleotide primers (5′ to 3′) were synthesized for amplification using Maximo Taq DNA Polymerase (GeneON GmbH, Ludwigshafen, Germany) and molecular oligonucleotide primers were purchased by Eurofins Genomics (Ebersberg, Germany). The forward and reverse DNA sequences of the primers utilized were (5′ to 3′): GGCCCTTCAYTAATTGGTGCT and GTGCATGACATGATGCTGGCT for NADH-plastoquinone oxidoreductase subunit 3 (*ndh*C) gene (845 bp), CCTTATCATTTAGAGGAAGGAG and TCCTCCGCTTATTGATATGC for ITS (720 bp; [99], CGCGCATGGTGGATTCACAATCC and GTTATGCATGAACGTAATGCTCfor *trn*H-*psb*A (411 bp; [100]) and ATTTGAACTGGTGACACGAG and CGAAATCGGTAGACGCTACG for *trn*L/*trn*F (879 bp; [101]). Annealing temperatures for primer pairs ranged from 55 °C to 62 °C, optimized for each primer set.

In total, 25 new DNA sequences from the examined tulip individuals were submitted to GenBank (https://www.ncbi.nlm.nih.gov/genbank/, accessed on 1 February 2026), and each was assigned a unique accession number.

Separate alignments were generated for each molecular marker examined in this study, using the MUSCLE algorithm. Specifically, the chloroplast genome markers *trn*L*/trn*F and *trn*H-*psb*A, together with the nuclear molecular marker ITS, were aligned with sequences of the *T. scardica* complex produced by previous studies [8] (GenBank accession numbers are provided in Table S2). The chloroplast marker *ndhC* was aligned with available *cp*DNA sequences retrieved from GenBank (GenBank accession numbers are provided in Table S2). A concatenated phylogenetic tree was constructed using the markers *trn*L/*trn*F, *trn*H-*psb*A, and ITS for specimens with sequences available for all three markers. In addition, a separate phylogenetic tree was constructed for *ndh*C. Substitution model selection was performed based on AIC values following model testing. Maximum-Likelihood trees were then generated with bootstrap support calculated for *n* = 100. The alignments of the above-mentioned sequences and phylogenetic tree construction were performed in MEGA 11.0 (Molecular Evolutionary Genetics Analysis) [102].

### 4.7. Analysis of Soil Properties

Composite surface soil samples (0–20 cm) were collected from the areas where the investigated wild-growing tulips were found. Specifically, four soil samples were collected, i.e., one sample from the region of Vytoumá where *T. scardica* was growing, and three samples from the regions of Dídima (two samples) and Emporiós (one sample) where *T. undulatifolia* populations were found. The soil samples were air-dried, passed through a 2 mm sieve and were analyzed in triplicate for the following properties. Soil size distribution was determined by the hydrometer method [103] and then soil texture classification was conducted. Organic carbon (C) was determined by the wet oxidation method [104], pH was measured in a 1:2 (*w*/*v*) water suspension and cation exchange capacity was assessed by the hexamminecobalt(III) chloride ([Co(NH_3_)_6_]Cl_3_) method [105]. In addition, the presence of calcium carbonate (CaCO_3_) was detected, by addition of a small quantity of 4 M hydrochloric acid (HCl) to soil, to observe or not the release of carbon dioxide (CO_2_) (bubbles).

As far as soil available macro- and micronutrients are concerned, both NO_3_-N and NH_4_-N were extracted with 1 M potassium chloride (KCl) and were measured with UV-Vis spectrometry and the sodium salicylate-sodium nitroprusside method, respectively [106]; P was extracted using 0.5 M NaHCO_3_, pH 8.5 and was measured by the molybdenum blue-ascorbic acid method [107]; K, Ca and Mg were extracted with 1 M ammonium acetate (CH_3_COONH_4_), pH 7 [108]; K was measured with flame photometry, while Ca and Mg were measured by atomic absorption spectrometry. The DTPA method [109] was used for Cu, Zn, Fe and Mn extractions, which were measured by atomic absorption spectrometry. Boron was extracted with hot water, and the determination was carried out with the azomethine-H method by UV-Vis spectrometry [110].

## 5. Conclusions

Based on the findings of the present study, *T. scardica* and *T. undulatifolia* are distinguishable by consistent differences in plant and floral morphology, encompassing both quantitative and qualitative traits, with further differentiation evident in seed morphology and karyotype structure, as well as habitat characteristics. Although DNA barcoding revealed no consistent genetic separation between the two taxa, previous studies have demonstrated the limited resolution of universal molecular markers in delimiting closely related species, highlighting the need for more targeted and taxon-specific molecular analyses. As far as the soil properties of *T. scardica* and *T. undulatifolia* are concerned, differences as well as similarities were evidenced. This was somewhat expected, since it is well known that the growth of only few plant species is strongly associated with soils with specific properties, such as sandy or acidic or saline soils. Taken together, the morphological, cytological, and ecological evidence indicates that the population discovered at the locality previously attributed to *T. scardica* differs in multiple aspects from *T. undulatifolia*. However, further studies including broader geographic sampling and higher-resolution molecular data will be necessary to more conclusively resolve the taxonomic status of *T. scardica* across its Balkan range. Unless future, broader, and more focused research demonstrates otherwise, *T. scardica* should be treated as a rediscovered species within the flora of Greece representing the southernmost known occurrence of this species to date.

## Figures and Tables

**Table 1:** Comparison of quantitative morphological traits (mean ± SD) measured in 25 individuals of *Tulipa scardica* and 115 individuals from four populations of *T. undulatifolia*. Different letters following the values indicate statistically significant differences among group means based on the Kruskal–Wallis post hoc test (*p* < 0.05).

Trait	*Tulipa scardica*	*Tulipa undulatifolia*
Plant Length (cm)	13.00 ± 5.41 a	23.86 ± 5.31 b
Stem Width (mm)	1.69 ± 0.22 a	2.32 ± 0.43 b
Length of Lowest Leaf (cm)	8.73 ± 1.52 a	17.46 ± 4.11 b
Width of Lowest Leaf (cm)	1.30 ± 0.19 a	3.35 ± 1.04 b
Length of Second Lowest Leaf (cm)	7.86 ± 1.50 a	16.92 ± 3.80 b
Width of Second Lowest Leaf (cm)	0.75 ± 0.15 a	2.18 ± 0.74 b
Length of Outer Tepal (cm)	3.21 ± 0.57 a	5.66 ± 0.82 b
Width of Outer Tepal (cm)	1.55 ± 0.35 a	2.12 ± 0.48 b
Blotch of Outer Tepal (cm)	0.77 ± 0.24 a	1.14 ± 0.35 b
Length of Inner Tepal (cm)	3.14 ± 0.49 a	5.45 ± 0.79 b
Width of Inner Tepal (cm)	1.71 ± 0.35 a	2.40 ± 0.48 b
Blotch of Inner Tepal (cm)	0.90 ± 0.21 a	1.33 ± 0.34 b
Length of Anther (mm)	4.48 ± 0.88 a	9.04 ± 2.51 b
Width of Anther (mm)	1.50 ± 0.34 a	2.88 ± 0.55 b
Length of Outer Filament (mm)	4.95 ± 0.97 a	7.06 ± 1.69 b
Length of Inner Filament (mm)	4.59 ± 1.15 a	6.59 ± 1.61 b
Length: Width of Lowest Leaf	6.84 ± 1.41 a	5.49 ± 1.28 b
Length: Width of Second Lowest Leaf	10.93 ± 2.97 a	8.29 ± 2.16 b
Length: Width of Outer Tepal	2.13 ± 0.47 a	2.76 ± 0.59 b
Length of Outer Tepal: Blotch of Outer Tepal	4.39 ± 1.07 a	5.30 ± 1.29 b
Length: Width of Inner Tepal	1.89 ± 0.37 a	2.33 ± 0.37 b
Length of Inner Tepal: Blotch of Inner Tepal	3.58 ± 0.61 a	4.25 ± 0.73 b
Length of Outer Tepal: Length of Inner Tepal	1.02 ± 0.06 a	1.04 ± 0.08 a
Width of Outer Tepal: Width of Inner Tepal	0.91 ± 0.12 a	0.89 ± 0.13 a
Length of Outer Filament: Length of Anther	1.13 ± 0.25 a	0.84 ± 0.31 b
Length of Inner Filament: Length of Anther	1.04 ± 0.26 a	0.79 ± 0.28 b

**Table 2:** Seed morphometric parameters measured for *Tulipa scardica* and *T. undulatifolia*. CPL indicates the length between the crossing point of (TL) and (TW) the nearest edge of the seed. Different letters following the values indicate statistically significant differences among group means based on the Kruskal–Wallis post hoc test (*p* < 0.05).

Trait	*Tulipa scardica*	*Tulipa undulatifolia*
Total length (mm)	5.82 ± 0.44 a	7.79 ± 0.58 b
Total width (mm)	4.99 ± 0.47 a	6.43 ± 0.52 b
Embryo length (mm)	1.85 ± 0.23 a	2.16 ± 0.38 b
Seed width (mm)	3.50 ± 0.38 a	4.75 ± 0.53 b
Wing width (mm)	1.31 ± 0.24 a	1.64 ± 0.28 b
Seed length (mm)	4.42 ± 0.40 a	5.91 ± 0.56 b
CPL (mm)	2.15 ± 0.34 a	2.70 ± 0.29 b
Embryo length/Seed length	0.42 ± 0.05 b	0.37 ± 0.08 a
Total length/Total width	1.17 ± 0.06 a	1.21 ± 0.07 b
Seed width/Wing width	2.74 ± 0.49 a	2.99 ± 0.66 a
Seed length/Seed width	1.27 ± 0.11 a	1.25 ± 0.11 a
Total length/CPL	2.75 ± 0.34 a	2.90 ± 0.19 b

**Table 3:** Karyomorphometric indices of the *Tulipa* species studied. Chromosome number (2*n*), karyotype formula, minimum (min l+s) and maximum (max l+s) chromosome length, total (TCL) and average (ACL) chromosome length, total haploid chromosome length (THL), karyotype asymmetry indices (CV_CL_ and M_CA_), and variation in centromeric position (CV_CI_) are presented. Values are given as mean ± standard deviation (SD). Statistically significant differences between species as determined by Welch’s *t*-test are indicated in bold. Significance levels are denoted as follows: * *p* < 0.05, *** *p* < 0.001. Effect sizes were calculated using Cohen’s d.

*Tulipa* Species	2*n*	Karyotype Formula	Min l+s (μm)	Max l+s (μm)	TCL (μm)	ACL (μm)	THL (μm)	CV_CL_	M_CA_	CV_CI_
*T. scardica*	24	8 sm + 4 sm-SAT + 2 sm/st-SAT + 10 st	8.54 (0.80)	13.91 (1.91)	266.07 (27.54)	11.09 (1.15)	133.04 (13.77)	15.92 (1.86) ***	45.73 (2.43)	24.95 (1.16) *
*T. undulatifolia*	24	12 sm + 4 sm-SAT + 6 sm/st + 2 st-SAT	8.53 (0.63)	15.05 (1.10)	275.35 (38.58)	11.47 (1.19)	137.67 (13.98)	19.22 (2.22) ***	45.67 (1.89)	23.07 (2.62) *

**Table 4:** Chromosome indices of the studied *Tulipa* species employing long arm’s length (l), short arm’s length (s), chromosome length (l+s), r-index (l/s), centromeric index, arm difference ratio, and R-length.

*Tulipa* Species	Chromosome Pair	l	s	l+s	l/s	Centromeric Index l/l+s	Arm Difference Ratio l-s/l+s	R-Length l+s/Sn(l+s)
*T.* *scardica* (2x)	1	10.02	3.89	13.91	2.86	0.716	0.432	0.052
	2	9.32	4.06	13.38	2.68	0.700	0.400	0.050
	3	9.44	3.52	12.96	2.94	0.731	0.463	0.049
	4	9.02	3.30	12.32	2.77	0.731	0.462	0.046
	5	8.80	2.84	11.64	3.27	0.753	0.506	0.044
	6	8.07	2.97	11.04	3.11	0.733	0.466	0.041
	7	7.48	3.11	10.59	2.48	0.705	0.410	0.040
	8	7.32	2.64	9.96	3.02	0.739	0.479	0.038
	9	6.87	3.10	9.97	2.27	0.689	0.378	0.038
	10	7.13	2.51	9.64	3.08	0.737	0.475	0.036
	11	6.73	2.34	9.07	3.06	0.744	0.489	0.034
	12	6.56	2.00	8.56	3.27	0.765	0.530	0.032
*T.* *undulatifolia* (2x)	1	10.53	4.52	15.05	2.71	0.701	0.402	0.055
	2	10.25	3.93	14.18	2.93	0.723	0.447	0.052
	3	9.92	3.69	13.61	2.88	0.729	0.457	0.049
	4	9.60	3.53	13.13	2.70	0.739	0.464	0.048
	5	9.15	3.15	12.31	3.02	0.744	0.487	0.045
	6	8.45	2.95	11.40	3.04	0.741	0.481	0.041
	7	7.97	2.74	10.71	3.00	0.743	0.485	0.039
	8	7.45	2.89	10.34	2.79	0.723	0.446	0.038
	9	7.36	2.57	9.93	3.02	0.741	0.482	0.036
	10	6.75	2.68	9.43	2.63	0.716	0.431	0.034
	11	6.60	2.45	9.05	2.89	0.732	0.465	0.033
	12	6.10	2.43	8.53	2.63	0.717	0.434	0.031

**Table 5:** General properties and available concentrations of macronutrients and micronutrients of the original soils of the studied wild-growing *Tulipa* species. Values represent means of three replications ± standard error (SE).

Soil Properties		*T. scardica*	*T. undulatifolia* (Dídima 1)	*T. undulatifolia* (Dídima 2)	*T. undulatifolia* (Emporiós)
Particle size distribution and characterization	Sand (%)	66 ± 0	78 ± 0	33 ± 0	27 ± 0
	Silt (%)	21 ± 0	8 ± 0	24 ± 0	27 ± 0
	Clay (%)	13 ± 0	14 ± 0	43 ± 0	46 ± 0
	Texture	Sandy loam	Sandy loam	Loam	Loam
	CEC (cmolc kg^−1^)	27.4 ± 1.1	30.6 ± 0.8	34.7 ± 0.1	39.8 ± 0.2
	pH (1:2 H_2_O)	7.4 ± 0.0	7.7 ± 0.1	7.5 ± 0.0	7.8 ± 0.0
	Organic C (%)	1.58 ± 0.18	1.21 ± 0.07	1.17 ± 0.04	0.64 ± 0.03
	Total N (%)	0.17 ± 0.01	0.21 ± 0.01	0.16 ± 0.00	0.09 ± 0.01
	C/N	12.1 ± 0.7	5.9 ± 0.0	7.1 ± 0.0	7.4 ± 0.0
Soil available macronutrients	NO_3_-N (mg kg^−1^)	3.2 ± 0.1	4.5 ± 0.4	8.0 ± 0.6	3.1 ± 0.1
	NH_4_-N (mg kg^−1^)	20.9 ± 0.8	27.1 ± 0.7	7.7 ± 2.2	8.1 ± 2.9
	P (mg kg^−1^)	15.0 ± 0.8	10.9 ± 2.4	4.2 ± 0.8	2.2 ± 0.1
	K (mg kg^−1^)	110 ± 16	593 ± 21	643 ± 6	585 ± 7
	Ca (mg kg^−1^)	1534 ± 293	3287 ± 69	4661 ± 160	4936 ± 81
	Mg (mg kg^−1^)	1234 ± 2	410 ± 11	479 ± 25	884 ± 0
Soil available micronutrients	B (mg kg^−1^)	0.25 ± 0.02	1.30 ± 0.11	0.77 ± 0.11	0.52 ± 0.07
	Cu (mg kg^−1^)	1.91 ± 0.02	3.38 ± 0.06	1.89 ± 0.05	2.05 ± 0.09
	Zn (mg kg^−1^)	0.49 ± 0.16	1.00 ± 0.01	0.71 ± 0.04	0.55 ± 0.00
	Fe (mg kg^−1^)	33.3 ± 1.1	11.6 ± 0.9	9.9 ± 0.1	8.6 ± 0.5
	Mn (mg kg^−1^)	35.6 ± 0.9	34.3 ± 1.0	32.2 ± 1.9	7.3 ± 0.1

**Table 6:** Key differences revealed by different approaches between *Tulipa scardica* and *T. undulatifolia*.

Category	Trait	*Tulipa scardica*	*Tulipa undulatifolia*
**Plant** **morphology**	Numerical morphometry	Small plants (13.0 ± 5.41 cm); longer leaves (lowest leaf length to width ratio 6.84 ± 1.41); wider tepals (2.13 ± 0.47); filament-to-anther ratio > 1	Bigger plants (23.86 ± 5.31 cm); wider leaves (lowest leaf length to width ratio 5.49 ± 1.28); longer tepals (2.76 ± 0.59); filament-to-anther ratio < 1
	Floral “waist”	Flowers not shaping a “waist”	Flowers shaping a “waist”
	Outer tepal shape and apex	Elliptic with acute to obtuse apex	Narrowly elliptic with acute to acuminate apex
	Inner tepal shape and apex	Obovate to spathulate with obtuse apex	Elliptic-ovate with acute apex
	Anthers	Yellowish or purplish	Yellow
	Pollen	Purplish	Greenish-yellow
**Seed** **morphology**	Seed size and embryo	Smaller seeds in size, smaller seed wings, more developed embryo	Bigger seeds in size, wider seed wings, less developed embryo
	Stomatal organization on seed coat	Two elongated guard cells surrounded by clearly differentiated subsidiary cells, forming a complete stomatal complex	Paired guard cells delimiting a narrow aperture, subsidiary cells indistinct, more symmetrical guard cells and less deeply sunken into the seed coat surface
**Karyotype**	Karyotype formula	2*n* = 2x = 8 sm + 4 sm-SAT + 2 sm/st-SAT + 10 st = 24	2*n* = 2x = 12 sm + 4 sm-SAT + 6 sm/st + 2 st-SAT = 24
	Chromosome sizes	8.54–13.91 μm	8.53–15.05 μm
	CV_CL_	15.92	19.22
	Satellite chromosomes	1–3 pairs; submetacentric (sm-SAT) or submetacentric/acrocentric (sm/st-SAT)	1–2 submetacentric (sm-SAT) or submetacentric/acrocentric (sm/st-SAT)
**DNA** **barcoding**		Close genetic relationship indicated by the cpDNA markers *trn*H-*psb*A, *trn*L/*trn*F and *ndh*C and the nuclear marker ITS. Phylogenetic analyses based on these markers do not provide consistent species-level resolution.	
**Habitat** **preferences**		Strictly associated with dry, rocky habitats with sparse natural vegetation	Primarily associated with abandoned agricultural land (occasionally on rocky slopes)
**Soil** **properties**		On calcareous soil (sandy loam in texture), with higher values of C/N ratio, adequate concentrations of available P, and inadequate to marginally sufficient concentrations of available K	Mainly on non-calcareous soils (loam in texture), with lower values of C/N ratio, lower (inadequate) concentrations of available P, and higher concentrations of available K

## Data Availability

The data supporting the findings of this study are included in the Supplementary Materials associated with this article. Any additional data or materials are available from the corresponding author upon reasonable request.

## Supplementary Materials

The following supporting information can be downloaded at: https://www.mdpi.com/article/10.3390/plants15091374/s1; Box S1. The chronicle of the rediscovery of *Tulipa scardica* in Greece; Box S2. *Tulipa undulatifolia* specimens examined morphologically; Table S1. Qualitative and quantitative traits used in the analysis. Trait types are indicated as QN (Quantitative) and QL (Qualitative). The table includes abbreviations, trait descriptions, and possible values for each character; Box S3. *Tulipa undulatifolia* specimens examined karyologically; Figure S1. Representative photos of individual seeds from *Tulipa scardica* and *T. undulatifolia*; Table S2. Accession numbers used for the phylogenetic analysis of *Tulipa* species. The accession numbers generated in the frame of the current study are indicated with an asterisk; Table S3. Nucleotide differences among the investigated *Tulipa* species resulted from the alignment of the sequences for the studied molecular marker ITS. Samples from four populations of *T. undulatifolia* were examined: U1—*T. undulatifolia* Dídima, Peloponnese (Samartza et al., 2024) [42]; U2—*T. undulatifolia* Agios Stefanos, Attica; U3—*T. undulatifolia* Fana, Chios Island; and U4—*T. undulatifolia* Emporiós, Chios Island. In addition, three samples from the studied population of *Tulipa* cf. *scardica* (S1, S2, and S3) were analyzed, together with S4, a taxonomically verified sample of *T. scardica* originating from North Macedonia [42]. The accession numbers (GenBank, NCBI) for each sequence are indicated in Table S2; Table S4. Nucleotide differences among the investigated *Tulipa* species resulted from the alignment of the fsequences for the studied molecular marker *psb*A-*trn*H. Samples from four populations of *T. undulatifolia* were examined: U1—*T. undulatifolia* Dídima, Peloponnese [42]; U2—*T. undulatifolia* Agios Stefanos, Attica; U3—*T. undulatifolia* Fana, Chios Island. In addition, three samples from the studied population of *Tulipa* cf. *scardica* (S2, S3) were analyzed, together with S4, a taxonomically verified sample of *T. scardica* originating from North Macedonia [42]. The accession numbers (GenBank, NCBI) for each sequence are indicated in Table S2; Table S5. Nucleotide differences among the investigated *Tulipa* species resulted from the alignment of the sequences for the studied molecular marker *trn*L-*trn*F. Samples from four populations of *T. undulatifolia* were examined: U1—*T. undulatifolia* Dídima, Peloponnese [42]; U2—*T. undulatifolia* Agios Stefanos, Attica; U3—*T. undulatifolia* Fana, Chios Island; and U4—*T. undulatifolia* Emporiós, Chios Island. In addition, three samples from the studied population of *T. scardica* (S1, S2, and S3) were analyzed, together with S4, a taxonomically verified sample of *T. scardica* originating from North Macedonia [42]. The accession numbers (GenBank, NCBI) for each sequence are indicated in Table S2; Table S6. Nucleotide differences among the investigated *Tulipa* species resulted from the alignment of the sequences for the studied molecular marker NADH-plastoquinone oxidoreductase subunit 3 (*ndhC* gene). Samples from four populations of *T. undulatifolia* were examined: U1—*T. undulatifolia* Dídima, Peloponnese [42]; U2—*T. undulatifolia* Agios Stefanos, Attica; U3—*T. undulatifolia* Fana, Chios Island; and U4—*T. undulatifolia* Emporiós, Chios Island. In addition, three samples from the studied population of *T. scardica* (S1, S2, and S3) were analyzed, together with S4, a taxonomically verified sample of *T. scardica* originating from North Macedonia [42]. The accession numbers (GenBank, NCBI) for each sequence are indicated in Table S2.
