# Supplementary material for: Taxonomic Reassessment and Rediscovery of Tulipa scardica Bornm. in Greece: Insights from Integrated Analyses Compared to T. undulatifolia Boiss
Source: Plants (Basel). 2026 Apr 30;15(9):1374. doi: 10.3390/plants15091374 (PMC13164589; doi:10.3390/plants15091374)
Supplement: Supplementary file 1 [file plants-15-01374-s001.zip › plants-4222214-supplementary.pdf]

Article

Taxonomic Reassessment and Rediscovery of *Tulipa scardica* Bornm. in Greece: Insights from Integrated Analyses Compared to *T. undulatifolia* Boiss.

Ioulietta Samartza [1,2], Eleni Kriemadi [1], Dimitris Pappas [3], Anastasia-Garyfallia Karagianni [4], Ioannis Kofinas [2], Theodora Matsi [4], Ioannis-Dimosthenis Adamakis [5], Georgios Tsoktouridis [2], Pepy Bareka [1,6,*] and Nikos Krigas [2,6,7,8,*]

1 Department of Crop Science, Agricultural University of Athens, Iera Odos 75, GR-11855 Athens, Greece; isamartza@elgo.gr (I.S.); ekriemadi@aua.gr (E.K.)
2 Institute of Plant Breeding and Genetic Resources, Hellenic Agricultural Organization—Dimitra (ELGO-Dimitra), P.O. Box 60458, GR-57001 Thessaloniki, Greece; kofinas.kallergis@gmail.com (I.K.); gtsok@elgo.gr (G.T.)
3 Institute of Nuclear & Radiological Sciences and Technology, Energy & Safety (INRASTES), National Centre for Scientific Research "Demokritos", GR-15310 Agia Paraskevi, Greece; d.pappas@ipta.demokritos.gr (D.P.)
4 Soil Science Laboratory, School of Agriculture, Aristotle University of Thessaloniki, GR-54124 Thessaloniki, Greece; anastasia-asia@hotmail.com (A.-G.K.); thmatsi@agro.auth.gr (T.M.)
5 Department of Biology, National and Kapodistrian University of Athens, GR-15784 Athens, Greece; iadamaki@biol.uoa.gr (I.-D.A.)
6 Wild Tulip Specialist Group, IUCN Species Survival Commission, International Union for the Conservation of Nature, IUCN Headquarters, Rue Mauverney 28, 1196 Gland, Switzerland
7 Institute of Olive Tree, Subtropical Crops and Viticulture, Hellenic Agricultural Organization—Dimitra (ELGO-Dimitra), GR-71307 Heraklion, Greece
8 Department of Agriculture, School of Agricultural Sciences, Hellenic Mediterranean University, GR-71410 Heraklion, Greece
* Correspondence: bareka@aua.gr (P.B.); nkrigas@elgo.gr (N.K.)

Supplementary Materials

Box S1. The chronicle of the rediscovery of *Tulipa scardica* in Greece**.**

When well-described in detail, old reports from botanists' expeditions in the past represent precious information that can be exploited in contemporary botanical expeditions. In this way, we decided to analyze all available information in combination with field research in order to recreate the path followed by P. Sintenis in May 1896 when he first collected the *Tulipa* specimen that was later taxonomically confirmed as *T. scardica* by K. Persson (see P. Sintenis's specimen number 1555 of 16-05-1896 deposited in the Herbarium of Lund (L!); http://herbarium.emg.umu.se/record1307482, accessed on 1 February 2026).

When Paul Ernst Emil Sintenis, a German botanist, pharmacist, and professional plant collector, reported his travels, impressions and findings in his correspondence with J. Dörfler in Vienna, Sintentis recorded that, during a six-day stay in 1896, he carried out several botanical excursions to Teizana, Agria, and Lechonia in Magnesia, all of which proved productive. On 1 May 1896, he reached Kalabaka – the western terminus of the old Thessalian Railway – for another botanical exploration, where he remained until mid-June. He noted that three vegetation zones—plains, foothills, and high mountains—converged in the region and undertook numerous excursions in all directions, resulting in the collection of many noteworthy plant specimens. Among these, *Colchicum haynaldii* Heuff. was collected on 14th May 1896 in a forest meadow near the monastery of Vytoumá ("Witomo" in Sintenis 1896:551), while a specimen identified by Sintenis as *Tulipa undulatifolia* Boiss. was collected the same day between Metochi and Vytoumá ('inter Metochi et Witomo, in frutisetis', see Sintenis 1896:1555).

As originally mentioned by P. Sintenis, quite close by in the original area there was the stone bridge of Sarakina; this bridge was always the crossing point to Vytoumá, Pili, and other villages of Kalambaka region

in Central Greece. Departing from Kalambaka in 1896 for his inventory, P. Sintenis as a genuine explorer would have crossed this stone bridge of Sarakina on his path to the Vytoumá Monastery. Recently, part of this stone bridge collapsed in September 2023 during a catastrophic flood occurring in the broader region (Photo exhibit S1). The original Sintenis's path most probably started in the village by the river and went uphill. Around this path, the vegetation is still dense and impassable alluring to the three vegetation zones and landscapes originally described by him in his letters to J. Dörfler in Vienna (referred to as an area with "plains, foothills, and high mountains").

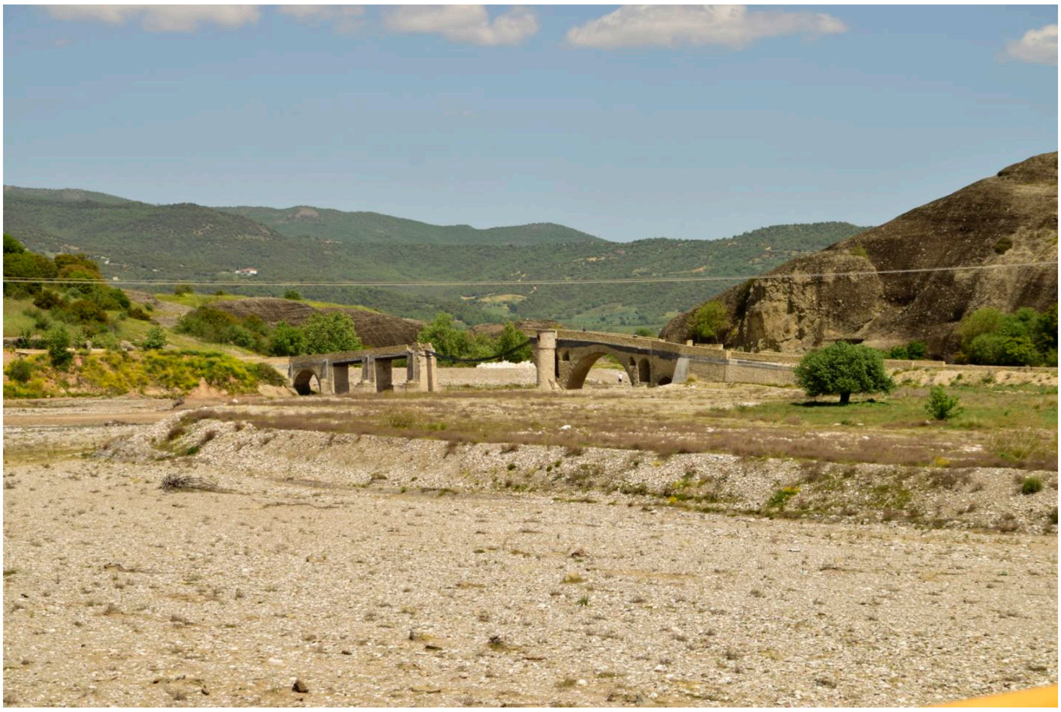

Photo exhibit S1. The landmark of the partially collapsed stone bridge of Sarakina in Kalambaka region (Central Greece) within the original landscape explored by P. Sintenis in 1896 (Photo by Ioannis Kofinas).

In the morning on 10 August 2024, as we were setting off to find the original location of *T. scardica* in Greece through the old Vytoumá path, we first tried to source information and precise indication by local inhabitants. When asked, some residents of the area mentioned that in the past they had seen wild tulips on the old path leading to the Vytoumá Monastery. According to the local testimonies, these tulips (presumed *T. scardica*) were not found in abandoned fields or cultivated lands like many other tulip species elsewhere in Greece, and such tulips were not to be found by anyone visiting the monastery from another road or path. It should be mentioned that the current road has no connection with this path from the village of Vytoumá to the local monastery because it is carved further south. Normally, this area should have been the original place where Sintenis first saw his *Tulipa* specimen and made his first tulip collection on May 14, 1896.
During this expedition, we arrived rather late in Vytoumá village to start exploring the area looking for wild tulips in fruiting stage. Realizing that we had only a few hours until nightfall, we preferred to immediately visit the village to ask locals at sheepfolds and local taverns. In our quest for information, they told us that there was indeed a small wild-growing tulip in their area, thus confirming the existence of a tulip in the area. The location referred to us by them was on the southwestern slope of the village towards the monastery; however, these tulips were not found in prominent places, but they were rather hidden in openings and ridges. Next day, we approached this location via dirt roads with this information in mind, and we found some fruiting tulip plants and noted their exact geographical coordinates to visit them again during their flowering period. With no flowering seen, the presence of *T. scardica* in the area could not be confirmed. Leaving for Pili later, we found another inhabitant (beekeeper) tending to his field with small olive trees.

When kindly asked by us, he remembered a different location with wild tulips south of Vytoumá, on the hill with Agios Georgios Chapel. According to this testimony, when his family and the local priest were going in this site to light the candles in the church, he remembered that he used to play with other children hide and seek in the bushes with wild tulips growing under them. We informed him that in such sites (mid-elevations) most tulips are normally expected to bloom at the end of April or beginning of May. He confirmed their seasonal blooming, and he told us that they were only found growing in the clearings and paths that climb the hill from the difficult-to-access side. Unfortunately, even though we visited this location, we did not find any sign of wild tulips. To verify the latter information, we decided to re-schedule further exploration for next spring.

During the Orthodox Easter period of 2025, we organized another botanical expedition in Vytoumá area. We arrived at the restaurant in the evening of 18 April 2025 for dinner where the locals remembered us immediately. The local beekeeper gave us even more details this time: "You will follow the old path to the Vytoumá Monastery; you will climb up to the monastery from the ridge for easy access and you will turn right towards the stream where the village is situated, and from there, you will enter the clearing of vegetation. Don't forget to move some branches aside, if necessary, when looking for tulips because these plants are hiding behind them. There are quite a few there; along the stream, you rarely see them scattered". We immediately remembered the reference to their existence along the trail.

We then used contemporary mountaineering maps, and we found out that the Vytoumá Monastery is connected at higher altitudes with the European E4 trail that passes through Diava at the Koromilia peak of Mt. Koziakas. Integrating all the above-mentioned information and combining with in-situ field research, we tried to recreate the path that P. Sintenis probably followed during his inventory in the area back in 1896 (Photo exhibit S2).

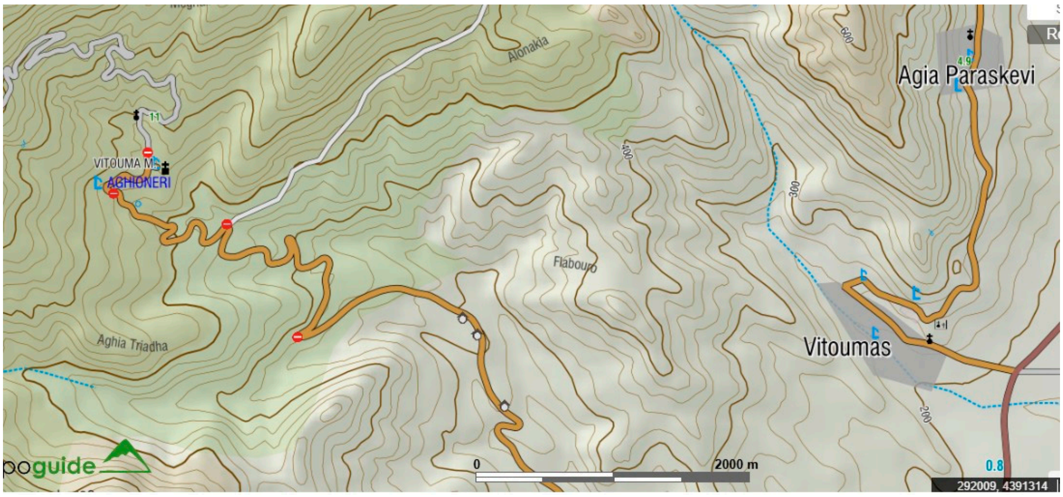

Photo exhibit S2. Recreation of the path most probably followed by P. Sintenis on his way to the area of Vytoumá Monastery based on local testimonies and field inventory.

In the morning of the day before Easter 2025 (Holly Saturday morning), on a flower-covered slope and ridge, as soon as we leaned over to the spot pointed out by the beekeeper, we found the autumn-flowering local Balkan endemic *Colchicum haynaldii* Heuff. with capsules, and right after, the large population of wild tulips with the first three individuals in bloom (Photo exhibit S3). Interestingly, Sintenis also collected *C. haynaldii* on the same day as the tulip, suggesting that the two species co-occurred in the area he explored (https://www.gbif.org/occurrence/788615196, accessed on 9 March 2026). The population was found in clearings of shrubby vegetation, mainly *Quercus coccifera* L. and *Juniperus oxycedrus* L. with phrygana on a northeast-facing slope, on serpentine rocky soils (Photo exhibit S4). There were limestone edges (Photo

exhibit S5) at the bottom of the hills as well as the ridges of Mt. Koziakas or Kerketio Mountain in Pindus mountain range (Kerketio is derived from Greek kerkída meaning stand; the local mountain standing over the Thessalian plain). In addition, some grey limestone rocks were present in the area, not at the base of the hills but rather at the edges and upper parts of the elevations. When reaching the ridge—that is, the upper part of the slope near the village—the terrain becomes relatively open and largely devoid of tulips. Likewise, the lower section along the old path, where the vegetation is very dense, contains only a few scattered individuals occurring in small openings. However, when moving to the right side of the ridge (a local slope crest rather than the main mountain ridgeline typical of larger mountains), the plants were found concealed within clearings of the shrubby vegetation. In these small openings, the tulips occur in patches, hidden among the surrounding shrubs.

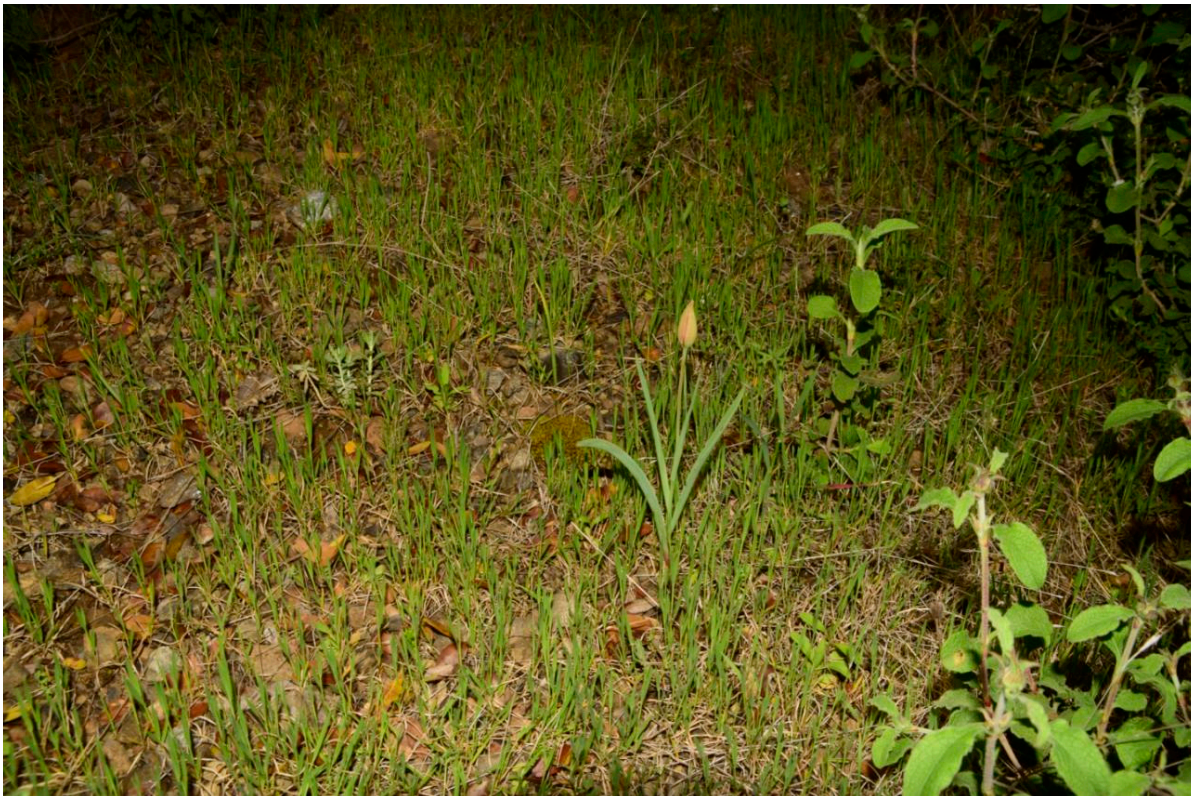

Photo exhibit S3. The first flowering plant of *Tulipa scardica* rediscovered in Greece in spring 2025 (Photo by Ioannis Kofinas).

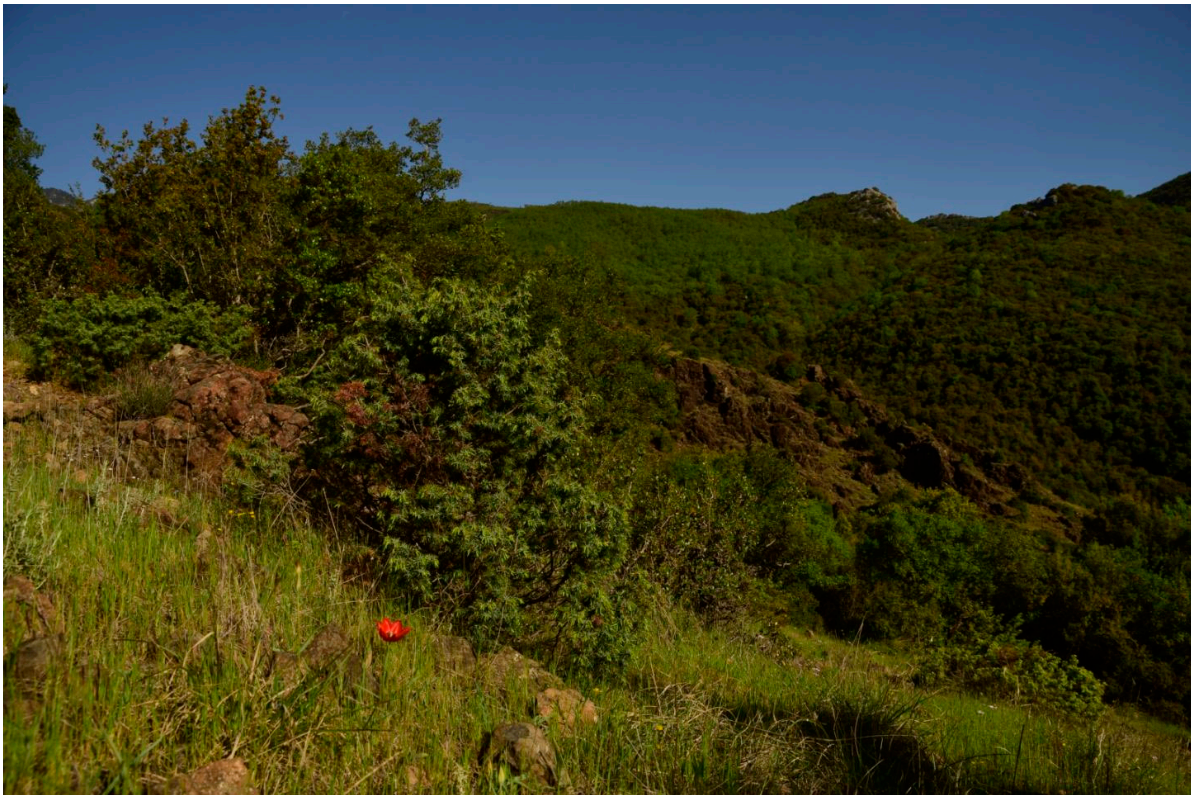

Photo exhibit S4. The original habitat of *Tulipa scardica* in Greece (Photo by Ioannis Kofinas).

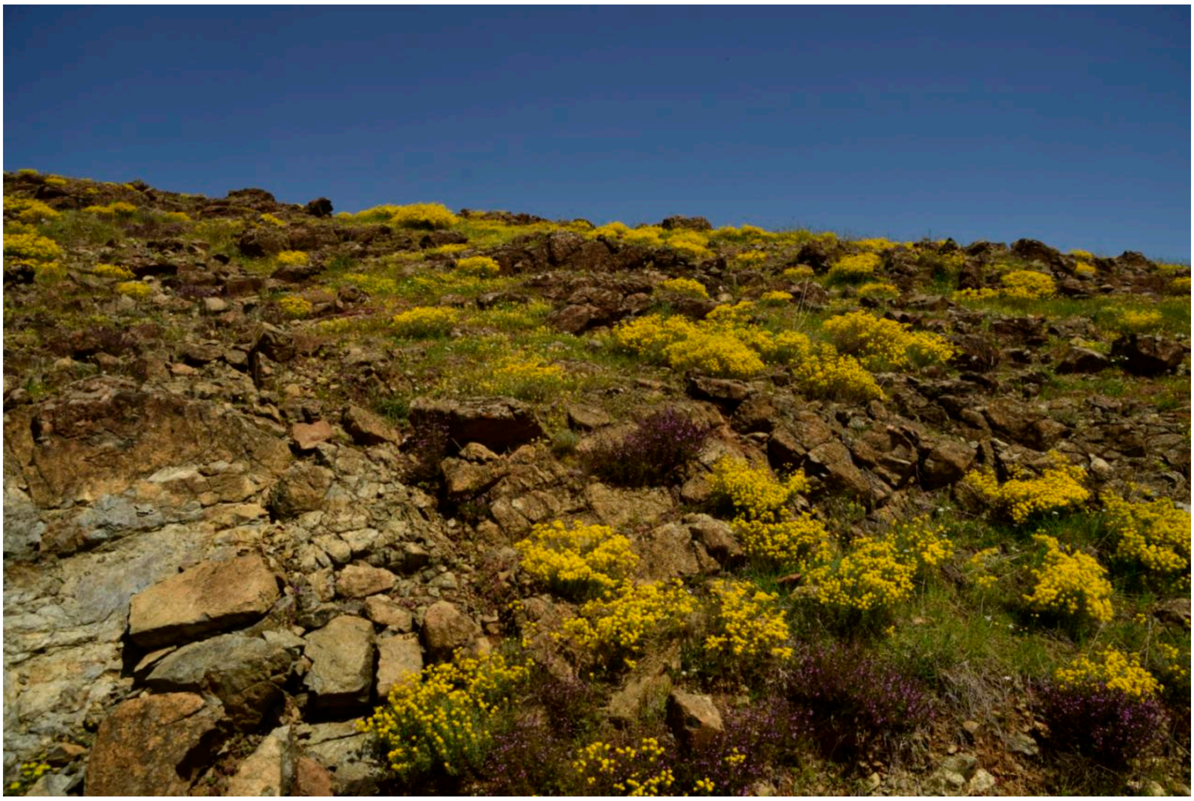

Photo exhibit S5. The original serpentine matrix along with limestone outcrops at the base of the hills where the Greek population of *Tulipa scardica* was found (Photo by Ioannis Kofinas).

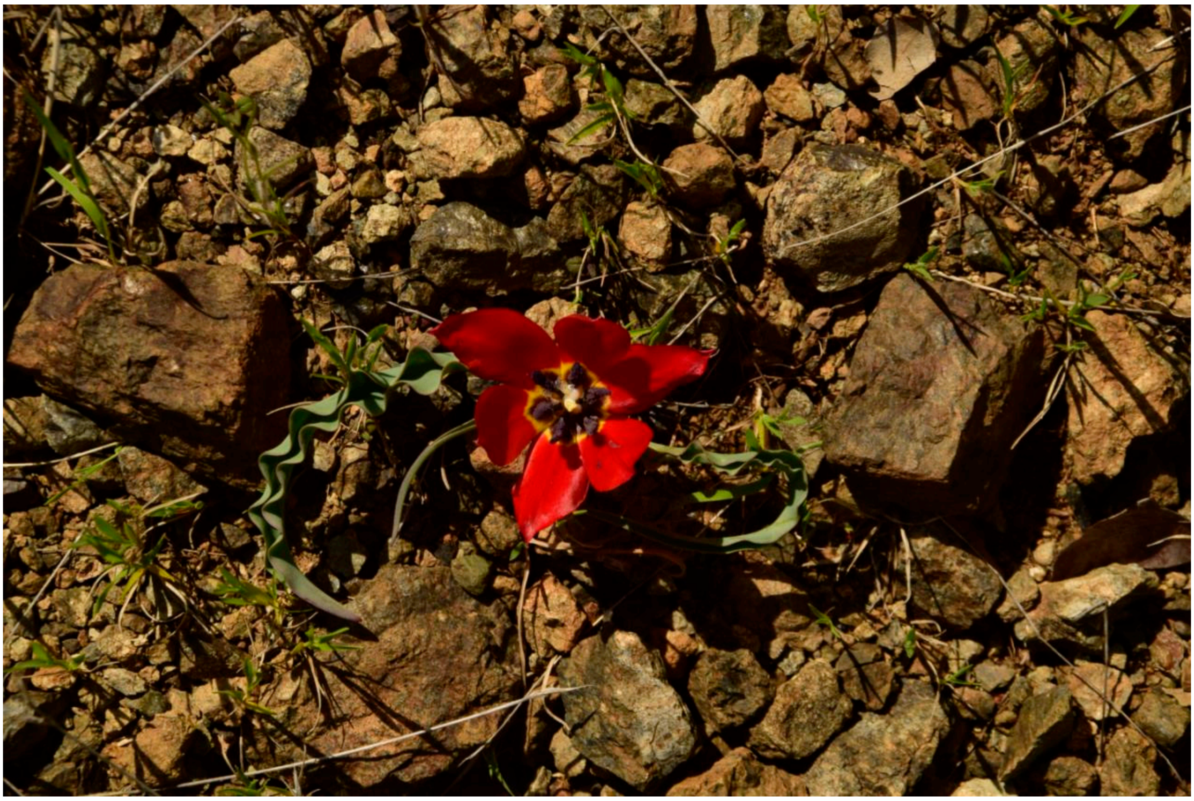

Photo exhibit S6. Vertical view of a *Tulipa scardica* flowering individual in its original locality in Greece (Photo by Ioannis Kofinas).

If Sintenis had never crossed that old path to the Vytoumá Monastery, this tulip population would have been known merely to local inhabitants, and no one else would have ever identified it taxonomically.

Box S2. *Tulipa undulatifolia* specimens examined morphologically

GREECE, Attica: East Attica Regional Unit, Agios Stefanos area, eastern foothills of Mount Parnitha, at margins of agricultural habitats, alt. 332.5 m, 38°09'47.0"N 23°52'13.0"E, 1 Apr 2025, I. Samartza, S. Papakonstantinou & G. Tsoktouridis GR-BBGK-1-25,449 (Herb. BBGK); **PELOPONNISOS, Argolis:** Didima Plain, Municipality of Ermionida, ex cultis, alt. 154.9 m, 37°27'01.3"N 23°10'38.3"E, 28 March 2025, I. Samartza, S. Papakonstantinou & G. Tsoktouridis GR-BBGK-1-25,130 (Herb. BBGK); **EAST AEGEAN ISLANDS, Chios Island:** Emporeios, olive groves, alt. 31.5 m, 38°12'11.4"N 26°01'47.9"E, 6 Apr 2025, I. Samartza & N. Krigas GR-BBGK-1-25,442 (Herb. BBGK); **Chios Island:** Fana, from Vatheia Lagkada towards Kato Fana, alt. 20.7 m, 38°12'38.9"N 25°56'08.0"E, 7 Apr 2025, I. Samartza & N. Krigas GR-BBGK-1-25,116 (Herb. BBGK).

Table S1. Qualitative and quantitative traits used in the analysis. Trait types are indicated as QN (Quantitative) and QL (Qualitative). The table includes abbreviations, trait descriptions, and possible values for each character.

Type	Abbreviation	Trait description	Value
QN	1-PL	Plant length	number
QN	4-SW	Stem width	number
QN	9-LLL	Length of lowest leaf	number
QN	10-WLL	Width of lowest leaf	number
QN	12-WSLL	Width of second lowest leaf	number
QN	15-LOT	Length of outer tepal	number
QN	16-WOT	Width of outer tepal	number
QN	18-LOT/BLOT	Length of outer tepal/ blotch length of outer tepal	number
QN	20-LOT/LIT	Length of outer tepal/ length of inner tepal	number
QN	21-WOT/WIT	Width of outer tepal/ width of inner tepal	number
QN	25-LIT/BLIT	Length of inner tepal/ blotch length of inner tepal	number
QN	29-LA	Length of anther	number
QN	30-WA	Width of anther	number
QN	31-LIF/LA	Length of inner filament/ length of anther	number
QN	32-LOF/LA	Length of outer filament/ length of anther	number
QL	1-SP	Stem pubescence	absent (0)/present (1)
QL	3-ES	Stolon presence	absent (0)/present (1)
QL	5-TT	Tunic type	papery (0)/coriaceus (leathery) (1)/crusty (2)
QL	6-TC	Tunic colour	brown or reddish-brown (0)/dark brown or blackish-brown(1)
QL	7-TTH	Type of tunic hairs	pilose (0)/lanate (1)
QL	8-OHM	Hair distribution and occurrence of hairs in the middle part of the tunic	absent (0)/present (1)
QL	12-LLT	Lowest leaf type	lanceolate (0)/linear-lanceolate (1)
QL	13-LLU	Lowest leaf undulation	absent (0)/present (1)
QL	17-SLLT	Second lowest leaf type	lanceolate (0)/linear-lanceolate (1)
QL	18-SLLU	Second lowest leaf undulation	absent (0)/present (1)
QL	22-FS	Flower shape	infundibular (0)/campanulate (1)
QL	24-BC	Blotch colour	absent (0)/yellow (1)/purple (2)/reddish-purple (3)/blurred purplish-black (4)/bicoloured (5)
QL	27-COTIS	Colour of outer tepal at inner surface	bright white (0)/bright yellow (1)/bright mauve (2)/bright pinkish-purple to magenta (3)/bright red (4)
QL	28-COTOS	Colour of outer tepal at outer surface	pale white tinged greyish-violet (0)/pale white with a reddish-pink band from the flower base to tip (1)/pale yellow tinged with greyish-violet (2)/ pale yellow tinged reddish-brown, reddish-pink, red or green (3)/pale mauve (4)/pale pinkish-purple to magenta (5)/pale red (6)

QL	29-SOT	Shape of outer tepal	narrowly elliptic (0)/elliptic (1)/elliptic-ovate to ovate (2)/elliptic-ovate to elliptic-obovate (3)
QL	30-TOT	Tip of outer tepal	acute to obtuse (0)/acute to acuminate (1)
QL	32-OTTP	Outer tepal tip pubescence	absent (0)/present (1)
QL	35-CITIS	Colour of inner tepal at inner surface	bright white (0)/bright yellow (1)/bright mauve (2)/bright pinkish-purple to magenta (3)/bright red (4)
QL	36-CITOS	Colour of inner tepal at outer surface	pale white (0)/pale yellow (1)/pale yellow often reddish-brown, reddishpink or green only at base (2)/pale mauve (3)/pale pinkish-purple to magenta (4)/pale-red (5)
QL	37-SIT	Shape of inner tepal	obovate to spathulate (0)/elliptic-ovate narrowed at base (1)
QL	38-TIT	Tip of inner tepal	obtuse (0)/acute (1)
QL	39-ITTP	Inner tepal tip pubescence	glabrous (0)/pubescent (1)
QL	43-FC	Filament colour	yellow to orange (0)/purple (1)/brownish-black to purplish-black (2)/ bicoloured (3)
QL	45-PFB	Pubescence of fillament base	absent (0)/present (1)

Box S3. *Tulipa undulatifolia* specimens examined karyologically.

GREECE, STEREA ELLAS, Attica: East Attica Regional Unit, Agios Stefanos area, eastern foothills of Mount Parnitha, at margins of agricultural habitats, alt. 289 m, 38°09'46''N, 23°52'02''E, 22 Apr 2022, E. Kriemadi & Chr. Kriemadi 1182 (Herb. ACA); **Phocis**: Desfina, Mesokampos area, along the provincial road Itea–Distomo, olive groves, alt. 506 m, 38°23'36''N, 22°35'08''E, 23 Apr 2023, E. Kriemadi & Chr. Kriemadi 1076 (Herb. ACA); **Phocis**: Desfina, Mesokampos area, along the provincial road Itea–Distomo, olive groves, alt. 506 m, 38°23'36''N, 22°35'08''E, 04 Apr 2024, E. Kriemadi & Chr. Kriemadi 1075 (Herb. ACA); **PELOPONNISOS: Argolis:** Didima Plain, Municipality of Ermionida, ex cultis, alt. 152 m, 37°27'09.399''N, 23°10'22.949''E, 20 May 2020, N. Krigas 1155 (Herb. ACA); **Argolis**: Didima Plain, Municipality of Ermionida, ex cultis, alt. 140–160 m, 37°27'11.0''N, 23°10'47.8''E, 25 Mar 2023, E. Kriemadi & S. Athanasiou 1156 (Herb. ACA); **WEST AEGEAN ISLANDS, Euboea:** Amarynthos, Agios Ioannis, area of ancient Tamynai (Tamynae) toponym, olive groves and vineyards, at the foothills of Mt. Kotylaio (offshoot of Mt. Dirfys), alt. 92 m, 38°26'35.0''N, 23°59'53.2''E, 13 Apr 2024, E. Kriemadi & Chr. Kriemadi 1074 (Herb. ACA); **EAST AEGEAN ISLANDS, Chios:** Fanos Cave, from Kato Fana towards Olympi Cave, alt. 117 m, 38°12'53''N, 25°55'31''E, 12 Apr 2022, E. Kriemadi & Chr. Kriemadi 1123 (Herb. ACA).

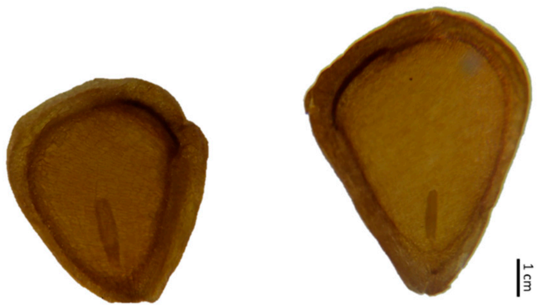

Figure S1. Representative photos of individual seeds from *Tulipa scardica* and *T. undulatifolia*.

Table S2. Accession numbers used for the phylogenetic analysis of *Tulipa* species. The accession numbers generated in the frame of the current study are indicated with an asterisk.

***Tulipa* species/Specimen**	**ITS**	***trn*H/*psb*A**	***trn*L/*trn*F**	***ndh*C**
T. albanica T1	MN336199	MZ147043	MN446897	–
T. albanica T2	MN336200	MZ147044	MN446898	–
T. albanica T3	MN336201	MZ147045	MN446899	–
T. kosovarica T4	MN336202	MZ147046	MN446900	–
T. kosovarica T5	MN336203	MZ147047	MN446901	–
T. kosovarica T6	MN336204	MZ147048	MN446902	–
T. kosovarica T7	MN336205	MZ147049	MN446903	–
T. kosovarica T8	–	MZ147050	MN446904	–
Tulipa species T9	MN336206	MZ147051	–	–
T. luanica T10	–	MZ147052	MN446905	–
T. luanica T11	MN336207	MZ147053	MN446906	–
T. luanica T12	MN336208	MZ147054	MN446907	–
T. luanica T13	MN336209	MZ147055	MN446908	–
T. luanica T14	MN336210	MZ147056	MN446909	–
T. scardica T15	MN336211	MZ147057	MN446910	–
T. scardica T16	MN336212	MZ147058	–	–
T. scardica T17	MN336213	MZ147059	–	–
T. serbica T18	MN336214	MZ147060	MN446911	–
T. serbica T19	MN336215	MZ147061	MN446912	–
T. serbica T20	MN336216	MZ147062	MN446913	–
T. scardica S1	PX965496*	–	PX965236*	PX965224*
T. scardica S2	PX965497*	PX965231*	PX965237*	PX965225*
T. scardica S3	PX965498*	PX965232*	PX965238*	PX965226*
T. scardica S4	OR206563	OR167012	PX965239*	PX965227*
T. undulatifolia U1	OR206561	OR167010	OR359615	PP934622*
T. undulatifolia U2	PX965499*	PX965234*	PX965240*	PX965230*
T. undulatifolia U3	PX965500*	PX965235*	PX965241*	PX965229*
T. undulatifolia U4	PX965501*	–	PX965242*	PX965228*
T. alberti	–	–	–	OR458821
T. altaica	–	–	–	NC_044780
T. buhseana	–	–	–	NC_052014

T. butkovii	–	–	–	NC_088749
T. dubia	–	–	–	NC_084336
T. fosteriana	–	–	–	NC_070244
T. gesneriana	–	–	–	NC_063831
T. greigii	–	–	–	PP338772
T. heterophylla	–	–	–	NC_088750
T. tianschanica	–	–	–	MW077738
T. iliensis	–	–	–	NC_052697
T. kaufmanniana	–	–	–	NC_087786
T. kolpakowskiana	–	–	–	NC_083129
T. korolkowii	–	–	–	PP693079
T. zenaidae	–	–	–	NC_086586
T. lemmersii	–	–	–	NC_086585
T. ostrowskiana	–	–	–	PP933988
T. sinkiangensis	–	–	–	NC_063595
T. schrenkii	–	–	–	NC_063594
T. sylvestris	–	–	–	MT261172
T. patens	–	–	–	NC_061194
T. brachystemon	–	–	–	NC_086584
T. tetraphylla	–	–	–	PP933989
T. zenaidae	–	–	–	NC_086586
T. zonneveldii	–	–	–	NC_088752

Table S3. Nucleotide differences among the investigated *Tulipa* species resulted from the alignment of the sequences for the studied molecular marker ITS. Samples from four populations of *T. undulatifolia* were examined: U1 - *T. undulatifolia* Didima, Peloponnese [42]; U2 - *T. undulatifolia* Agios Stefanos, Attica; U3 - *T. undulatifolia* Fana, Chios Island; and U4 - *T. undulatifolia* Emporeios, Chios Island. In addition, three samples from the studied population of *Tulipa* cf. *scardica* (S1, S2, and S3) were analyzed, together with S4, a taxonomically verified sample of *T. scardica* originating from North Macedonia [42]. The accession numbers (GenBank, NCBI) for each sequence are indicated in Table S2.

**Species and specimen**	**Locus**	
	**543**	**556**
***T. albanica* T1**	T	A
***T. albanica* T2**	T	A
***T. albanica* T3**	Y	R
***T. kosovarica* T4**	C	G
***T. kosovarica* T5**	C	R
***T. kosovarica* T6**	Y	R
***T. kosovarica* T7**	C	R
***Tulipa* species T9**	C	R
***T. luanica* T11**	C	R
***T. luanica* T12**	C	R
***T. luanica* T13**	Y	R
***T. luanica* T14**	C	R
***T. scardica* T15**	C	R
***T. scardica* T16**	C	R
***T. scardica* T17**	C	R
***T. serbica* T18**	C	R

***T. serbica* T19**	Y	R
***T. serbica* T20**	C	R
***T. scardica* S1**	C	G
***T. scardica* S2**	C	A
***T. scardica* S3**	C	G
***T. scardica* S4**	C	G
***T. undulatifolia* U1**	C	G
***T. undulatifolia* U2**	C	A
***T. undulatifolia* U3**	C	A
***T. undulatifolia* U4**	C	A

Table S4. Nucleotide differences among the investigated *Tulipa* species resulted from the alignment of the sequences for the studied molecular marker *psb*A-*trn*H. Samples from four populations of *T. undulatifolia* were examined: U1 - *T. undulatifolia* Didima, Peloponnese [42]; U2 - *T. undulatifolia* Agios Stefanos, Attica; U3 - *T. undulatifolia* Fana, Chios Island. In addition, three samples from the studied population of *Tulipa cf. scardica* (S2, S3) were analyzed, together with S4, a taxonomically verified sample of *T. scardica* originating from North Macedonia [42]. The accession numbers (GenBank, NCBI) for each sequence are indicated in Table S2.

	**Locus**						
**Species and specimen**	**24**	**60**	**93**	**95**	**96**	**98**	**380**
***T. albanica* T1**	A	C	C	A	G	A	G
***T. albanica* T2**	A	C	C	A	G	A	G
***T. albanica* T3**	A	C	C	A	G	A	G
***T. kosovarica* T4**	A	C	T	C	T	G	G
***T. kosovarica* T5**	A	C	T	C	T	G	G
***T. kosovarica* T6**	A	C	T	C	T	G	G
***T. kosovarica* T7**	A	C	T	C	T	G	G
***T. kosovarica* T8**	A	C	T	C	T	G	G
***Tulipa* species T9**	A	C	T	C	T	G	G
***T. luanica* T10**	A	C	T	C	T	G	G
***T. luanica* T11**	A	C	T	C	T	G	G
***T. luanica* T12**	A	C	T	C	T	G	G
***T. luanica* T13**	A	C	T	C	T	G	G
***T. luanica* T14**	A	C	T	C	T	G	G
***T. scardica* T15**	A	C	T	C	T	G	G
***T. scardica* T16**	A	C	T	C	T	G	G
***T. scardica* T17**	A	C	T	C	T	G	G
***T. serbica* T18**	A	C	T	C	T	G	G
***T. serbica* T19**	A	C	T	C	T	G	G
***T. serbica* T20**	A	C	T	C	T	G	G
***T. scardica* S2**	A	C	T	C	T	G	G
***T. scardica* S3**	A	C	T	C	T	G	G
***T. scardica* S4**	A	C	T	C	T	G	G
***T. undulatifolia* U1**	A	T	T	C	T	G	T
***T. undulatifolia* U2**	-	T	T	C	T	G	T
***T. undulatifolia* U3**	-	C	T	C	T	G	G

Table S5. Nucleotide differences among the investigated *Tulipa* species resulted from the alignment of the sequences for the studied molecular marker *trn*L-*trn*F. Samples from four populations of *T. undulatifolia* were examined: U1 - *T. undulatifolia* Didima, Peloponnese [42]; U2 - *T. undulatifolia* Agios Stefanos, Attica; U3 - *T. undulatifolia* Fana, Chios Island; and U4 - *T. undulatifolia* Emporeios, Chios Island. In addition, three samples from the studied population of *T. scardica* (S1, S2, and S3) were analyzed, together with S4, a taxonomically verified sample of *T. scardica* originating from North Macedonia [42]. The accession numbers (GenBank, NCBI) for each sequence are indicated in Table S2.

	**Locus**							
**Species and specimen**	**113-122**	**290**	**319**	**344**	**508**	**623**	**679**	**707-711**
***T. albanica* T1**	---------A	T	A	G	-	T	T	-----
***T. albanica* T2**	---------A	T	A	G	-	T	T	-----
***T. albanica* T3**	---------A	T	A	G	-	T	T	-----
***T. kosovarica* T4**	---------A	G	A	A	-	T	T	---AA
***T. kosovarica* T5**	---------A	G	A	A	-	T	T	----A
***T. kosovarica* T6**	--------AA	G	A	A	-	T	G	----A
***T. kosovarica* T7**	--------AA	G	A	A	-	T	G	----A
***T. kosovarica* T8**	--------AA	G	A	A	-	T	G	----A
***T. luanica* T10**	----------	G	A	A	-	T	T	---AA
***T. luanica* T11**	---------A	G	A	A	-	T	T	----A
***T. luanica* T12**	---------A	G	A	A	-	T	T	----A
***T. luanica* T13**	---------A	G	A	A	-	T	T	----A
***T. luanica* T14**	---------A	G	A	A	-	T	T	----A
***T. scardica* T15**	----AAAAAA	T	T	G	-	T	T	----A
***T. serbica* T16**	---------A	G	A	A	-	T	T	----A
***T. serbica* T19**	---------A	G	A	A	-	T	T	----A
***T. serbica* T20**	---------A	G	A	A	-	T	T	----A
***T. scardica* S1**	----------	T	A	G	A	T	T	-----
***T. scardica* S2**	----------	T	A	G	A	T	T	-----
***T. scardica* S3**	----------	T	A	G	A	T	T	-----
***T. scardica* S4**	AAAAAAAAAA	T	A	G	A	-	T	-----
***T. undulatifolia* U1**	----------	T	A	G	A	T	T	--AAA
***T. undulatifolia* U2**	----------	T	A	G	A	T	T	AAAAA
***T. undulatifolia* U3**	----------	T	A	G	A	T	T	----A
***T. undulatifolia* U4**	----------	T	A	G	A	T	T	----A

Table S6. Nucleotide differences among the investigated *Tulipa* species (and specimens) resulted from the alignment of the sequences for the studied molecular marker NADH-plastoquinone oxidoreductase subunit 3 (*ndhC* gene). Samples from four populations of *T. undulatifolia* were examined: U1 - *T. undulatifolia* Didima, Peloponnese [42]; U2 - *T. undulatifolia* Agios Stefanos, Attica; U3 - *T. undulatifolia* Fana, Chios Island; and U4 - *T. undulatifolia* Emporeios, Chios Island. In addition, three samples from the studied population of *T. scardica* (S1, S2, and S3) were analyzed, together with S4, a taxonomically verified sample of *T. scardica* originating from North Macedonia [42]. The accession numbers (GenBank, NCBI) for each sequence is indicated in Table S2.

Species	Locus																			
	**3**	**8**	**10**	**26**	**38**	**68**	**77**	**137-149**	**170**	**185**	**195-209**	**211**	**216**	**238**	**280**	**301**	**307**	**311-314**	**337**	**348**
T. alberti	C	C	C	T	C	G	C	-------------	T	G	---------------	T	T	G	A	T	A	ATTC	T	C
T. altaica	C	C	C	T	C	G	C	-------------	T	G	---------------	T	C	G	G	T	A	ATTC	G	C
T. buhseana	C	C	C	T	C	G	C	-------------	T	G	---------------	T	C	G	G	T	A	ATTC	G	C
T. butkovii	C	C	C	T	C	G	C	-------------	T	G	---------------	T	C	G	A	T	A	ATTC	T	C
T. dubia	C	C	C	T	C	G	C	-------------	T	G	---------------	T	C	G	A	T	A	ATTC	T	C
T. fosteriana	C	C	C	T	C	G	C	-------------	T	G	TTCATCCATAACTTC	T	C	A	G	T	A	ATTC	G	C
T. gesneriana	C	C	C	T	C	G	C	-------------	T	G	TTCATCCATAACTTC	T	C	A	G	T	A	ATTC	G	C
T. greigii	C	C	C	T	C	G	C	-------------	T	G	---------------	T	C	G	A	T	A	ATTC	T	C
T. heterophylla	T	C	C	C	C	T	C	TAATTTAATATAA	T	G	---------------	T	C	G	G	G	C	ATTC	G	T
T. tianschanica	C	C	T	T	C	G	C	-------------	T	G	---------------	T	C	G	G	G	A	ATTC	G	C
T. iliensis	C	C	C	T	C	G	C	-------------	T	G	---------------	T	C	G	G	T	A	ATTC	G	C
T. kaufmanniana	C	C	C	T	C	G	C	-------------	T	G	---------------	T	T	G	A	T	A	ATTC	T	C
T. kolpakowskiana	C	C	C	T	C	G	C	-------------	T	G	---------------	T	C	G	G	G	A	ATTC	G	C
T. korolkowii	C	C	C	T	C	G	C	-------------	T	G	---------------	T	C	G	G	G	A	ATTC	G	C
T. zenaidae	C	C	C	C	C	T	C	-------------	T	G	---------------	T	C	G	G	T	A	ATTC	G	T
T. lemmersii	C	C	C	T	A	G	C	-------------	T	G	---------------	T	C	G	G	G	A	ATTC	G	C
T. ostrowskiana	C	C	C	T	C	G	C	-------------	T	G	---------------	T	C	G	G	G	A	ATTC	G	C
***T. scardica* S1**	C	C	C	T	C	G	C	-------------	T	G	TTCATCCATAACTTC	T	C	A	G	T	A	ATTC	G	C
***T. scardica* S2**	C	C	C	T	C	G	C	-------------	T	G	TTCATCCATAACTTC	T	C	A	G	T	A	ATTC	G	C
***T. scardica* S3**	C	C	C	T	C	G	C	-------------	T	G	TTCATCCATAACTTC	T	C	A	G	T	A	ATTC	G	C
***T. scardica* S4**	C	C	C	T	C	G	C	-------------	T	G	TTCATCCATAACTTC	T	C	A	G	T	A	ATTC	G	C
T. sinkiangensis	C	C	C	C	C	G	C	-------------	G	G	---------------	T	C	G	G	T	A	----	G	C
T. schrenkii	C	C	C	T	C	G	C	-------------	T	G	---------------	T	C	G	G	G	A	ATTC	G	C
T. sylvestris	C	C	C	T	C	G	C	-------------	T	G	---------------	C	C	G	G	T	A	ATTC	G	C
T. patens	C	C	C	T	C	G	C	-------------	T	G	---------------	T	C	G	G	T	A	ATTC	G	C
T. brachystemon	C	C	C	T	C	G	C	-------------	T	G	---------------	T	C	G	G	G	A	ATTC	G	C
T. tetraphylla	C	C	C	T	C	G	C	-------------	T	G	---------------	T	C	G	G	T	A	ATTC	G	C
***T. undulatifolia* U1**	C	C	C	T	C	G	C	-------------	T	G	TTCATCCATAACTTC	T	C	A	G	T	A	ATTC	G	C
***T. undulatifolia* U2**	C	C	C	T	C	G	T	-------------	T	G	TTCATCCATAACTTC	T	C	A	G	T	A	ATTC	G	C
***T. undulatifolia* U3**	C	C	C	T	C	G	T	-------------	T	G	TTCATCCATAACTTC	T	C	A	G	T	A	ATTC	G	C
***T. undulatifolia* U4**	C	C	C	T	C	G	C	-------------	T	G	TTCATCCATAACTTC	T	C	A	G	T	A	ATTC	G	C
T. zenaidae	C	T	C	T	A	G	C	-------------	T	G	---------------	T	C	G	G	G	A	ATTC	G	C
T. zonneveldii	C	C	C	T	C	G	C	-------------	T	A	---------------	T	C	G	A	T	A	ATTC	T	C

**Species**	**Locus**												
	**366**	**367**	**372**	**395**	**411**	**452-464**	**478-497**	**521**	**525**	**560-562**	**568-574**	**592**	**596-605**
T. alberti	A	C	A	C	G	-------------	TATAGTA-------------	G	T	---	------T	C	----------
T. altaica	A	C	A	C	G	-------------	TATAGTA-------------	G	G	---	----TTT	C	----------
T. buhseana	A	C	G	C	G	TATCTA-------	--------------------	G	G	---	-----TT	C	----------
T. butkovii	A	C	A	C	G	-------------	TATAGTA-------------	G	T	---	------T	C	----------
T. dubia	A	C	A	C	G	-------------	TATAGTA-------------	G	T	---	------T	C	----------
T. fosteriana	A	C	A	C	G	-------------	TATAGTA-------------	G	G	---	-TTTTTT	C	----------
T. gesneriana	A	C	A	C	G	-------------	TATAGTA-------------	G	G	---	-TTTTTT	C	----------
T. greigii	A	C	A	C	G	-------------	TATAGTA-------------	G	T	---	------T	C	----------
T. heterophylla	A	C	G	C	G	TATCTATATCTAG	TATAGTATGTCTATATATAG	C	G	---	-------	A	----------
T. tianschanica	C	C	G	C	G	-------------	--------------------	G	G	---	------T	C	----------
T. iliensis	A	C	A	C	G	-------------	TATAGTA-------------	G	G	---	---TTTT	C	----------
T. kaufmanniana	A	C	A	C	G	-------------	TATAGTA-------------	G	T	---	------T	C	----------
T. kolpakowskiana	C	C	G	C	G	-------------	--------------------	G	G	---	------T	C	----------
T. korolkowii	C	C	G	C	G	-------------	--------------------	G	G	---	------T	C	----------
T. zenaidae	A	C	G	C	G	-------------	--------------------	G	G	TAT	TTTTTTT	C	----------
T. lemmersii	C	C	G	C	G	-------------	--------------------	G	G	---	------T	C	----------
T. ostrowskiana	C	C	G	C	G	-------------	--------------------	G	G	---	------T	C	----------
***T. scardica* S1**	A	C	A	C	G	-------------	TATAGTA-------------	G	G	---	---TTTT	C	----------
***T. scardica* S2**	A	C	A	C	G	-------------	TATAGTA-------------	G	G	---	----TTT	C	----------
***T. scardica* S3**	A	C	A	C	G	-------------	TATAGTA-------------	G	G	---	----TTT	C	----------
***T. scardica* S4**	A	C	A	C	G	-------------	TATAGTA-------------	G	G	---	---TTTT	C	TTAATATAAA
T. sinkiangensis	A	T	G	T	A	-------------	--------------------	G	G	---	-------	C	----------
T. schrenkii	C	C	G	C	G	-------------	--------------------	G	G	---	------T	C	----------
T. sylvestris	A	C	G	C	G	-------------	--------------------	G	G	---	TTTTTTT	C	----------
T. patens	A	C	G	C	G	-------------	--------------------	G	G	---	-----TT	C	----------
T. brachystemon	C	C	G	C	G	-------------	--------------------	G	G	---	------T	C	----------
T. tetraphylla	A	C	G	C	G	-------------	--------------------	G	G	---	-------	C	----------
***T. undulatifolia* U1**	A	C	A	C	G	-------------	TATAGTA-------------	G	G	---	----TTT	C	----------
***T. undulatifolia* U2**	A	C	A	C	G	-------------	TA------------------	G	G	---	------T	C	----------
***T. undulatifolia* U3**	A	C	A	C	G	-------------	TA------------------	G	G	---	------T	C	----------
***T. undulatifolia* U4**	A	C	A	C	G	-------------	TATAGTA-------------	G	G	---	----TTT	C	----------
T. zenaidae	C	C	G	C	G	-------------	--------------------	G	G	---	------T	C	----------
T. zonneveldii	A	C	A	C	G	-------------	TATAGTA-------------	G	T	---	------T	C	----------

Species	Locus										
	621	654	657-661	670	728	730	755-760	791	792-803	806	884
T. alberti	-	G	-----	C	G	T	------	G	CAAAA----AG	G	n
T. altaica	-	G	TAATT	C	G	T	------	G	CAAAA----AA	G	n
T. buhseana	-	A	TAATT	C	G	T	CTAAAA	G	CAAAAAAGCAG	G	n
T. butkovii	-	G	-----	C	G	T	------	G	CAAAA----AG	G	n
T. dubia	-	G	-----	C	G	T	------	G	CAAAA----AG	G	n
T. fosteriana	-	G	TAATT	C	G	C	------	G	CAAAA----AG	G	n
T. gesneriana	-	G	TAATT	C	G	C	------	G	CAAAA----AG	G	n
T. greigii	-	G	-----	C	G	T	------	G	CAAAA----AG	G	n
T. heterophylla	-	G	TAATT	C	G	T	------	G	---------AG	G	n
T. tianschanica	-	G	TAATT	C	G	T	------	G	CAAAA----AG	A	n
T. iliensis	-	G	TAATT	C	G	T	------	G	CAAAA----AA	G	n
T. kaufmanniana	-	G	-----	C	G	T	------	G	CAAAA----AG	G	n
T. kolpakowskiana	-	G	TAATT	C	G	T	------	G	CAAAA----AG	A	n
T. korolkowii	-	G	TAATT	C	G	T	------	G	CAAAA----AG	A	n
T. zenaidae	G	G	TAATT	T	G	T	------	G	CAAAAAAGCAG	G	n
T. lemmersii	-	G	TAATT	C	A	T	------	G	CAAAA----AG	A	n
T. ostrowskiana	-	G	TAATT	C	G	T	------	G	CAAAA----AG	A	n
***T. scardica* S1**	-	G	TAATT	C	G	C	------	G	CAAAA----AG	G	T
***T. scardica* S2**	-	G	TAATT	C	G	C	------	G	CAAAA----AG	G	T
***T. scardica* S3**	-	G	TAATT	C	G	C	------	G	CAAAA----AG	G	T
***T. scardica* S4**	-	G	TAATT	C	G	C	------	G	CAAAA----AG	G	-
T. sinkiangensis	-	G	TAATT	C	G	T	------	A	CAAAAAAGCAG	G	n
T. schrenkii	-	G	TAATT	C	G	T	------	G	CAAAA----AG	A	n
T. sylvestris	-	G	TAATT	C	G	T	------	G	CAAAAAAGCAG	G	n
T. patens	-	G	TAATT	C	G	T	------	G	CAAAAAAGCAG	G	n
T. brachystemon	-	G	TAATT	C	G	T	------	G	CAAAA----AG	A	n
T. tetraphylla	-	G	TAATT	C	G	T	------	G	CAAAA----AG	A	n
***T. undulatifolia* U1**	-	G	TAATT	C	G	C	------	G	CAAAA----AG	G	T
***T. undulatifolia* U2**	-	G	TAATT	C	G	C	------	G	CAAAA----AG	G	T
***T. undulatifolia* U3**	-	G	TAATT	C	G	C	------	G	CAAAA----AG	G	T
***T. undulatifolia* U4**	-	G	TAATT	C	G	C	------	G	CAAAA----AG	G	T
T. zenaidae	-	G	TAATT	C	G	T	------	G	CAAAA----AG	A	n
T. zonneveldii	-	G	-----	C	G	T	------	G	CAAAA----AG	G	n
